# Supplementary material for: Diagnostic value of the antiglycoprotein-2 antibody for Crohn’s disease: a PRISMA-compliant systematic review and meta-analysis
Source: BMJ Open. 2017 Jun 9;7(6):e014843. doi: 10.1136/bmjopen-2016-014843 (PMC5734552; doi:10.1136/bmjopen-2016-014843)
Supplement: Supplementary material 1 [file bmjopen-2016-014843supp001.pdf]

The specific search algorithms of Pubmed

((anti-glycoprotein[All Fields] AND 2[All Fields] AND ("immunoglobulins"[MeSH Terms] OR "immunoglobulins"[All Fields] OR "antibody"[All Fields] OR "antibodies"[MeSH Terms] OR "antibodies"[All Fields])) OR (("glycoproteins"[MeSH Terms] OR "glycoproteins"[All Fields] OR "glycoprotein"[All Fields]) AND 2[All Fields] AND ("autoantibodies"[MeSH Terms] OR "autoantibodies"[All Fields])) OR (("autoantibodies"[MeSH Terms] OR "autoantibodies"[All Fields]) AND ("glycoproteins"[MeSH Terms] OR "glycoproteins"[All Fields] OR "glycoprotein"[All Fields]) AND 2[All Fields])) AND ("crohn disease"[MeSH Terms] OR ("crohn"[All Fields] AND "disease"[All Fields]) OR "crohn disease"[All Fields] OR ("crohn's"[All Fields] AND "disease"[All Fields]) OR "crohn's disease"[All Fields])
