# Supplementary material for: Diagnostic value of the antiglycoprotein-2 antibody for Crohn’s disease: a PRISMA-compliant systematic review and meta-analysis
Source: BMJ Open. 2017 Jun 9;7(6):e014843. doi: 10.1136/bmjopen-2016-014843 (PMC5734552; doi:10.1136/bmjopen-2016-014843)
Supplement: Supplementary Table 1 [file bmjopen-2016-014843supp002.pdf]

Supplementary Table 1 Characteristics of studies included in the meta-analysis of diagnostic performance of anti-glycoprotein 2 antibody in CD

Reference	Year	Region	Author	Manufacturer	Method	Ig subtype	Case	Control	TP	FP	FN	TN	QUADAS score
						IgG			58	7	154	242	
[6]	2016	United Kingdom	Pavlidis et al.	In-house kit	IIF	IgA	212	249	21	2	191	247	12
						either			59	8	153	241	
						IgG			35	0	189	136	
[7]	2015	Germany	Michaels et al.	Euroimmune	IIF	IgA	224	136	23	4	201	132	12
						either			42	4	182	132	
						IgG			8	11	65	158	
[8]	2015	Germany	Roggenbuck et al.	GA Generic Assays	ELISA	IgA	73	169	19	8	54	161	11
						either			25	19	48	150	
						IgG			14	7	21	49	
[9]	2015	China	Zhang et al.	GA Generic Assays	ELISA	IgA	35	56	13	2	22	54	11
						either			19	9	16	47	
						IgG	323	509	87	15	236	494	
[10]	2015	United Kingdom	Pavlidis et al.	Inova Diagnostics	ELISA	IgA			48	10	275	499	12
						either	323	294	99	13	224	281	
						IgG			8	2	166	124	
[11]	2015	Germany	Laass et al.	GA Generic Assays	ELISA	IgA	174	129	34	7	140	122	10
						either			35	9	139	117	

Supplementary Table 1 Characteristics of studies included in the meta-analysis of diagnostic performance of anti-glycoprotein 2 antibody in CD (continued)

Reference	Year	Region	Author	Manufacturer	Method	Ig subtype	Case	Control	TP	FP	FN	TN	QUADAS score
[12]	2015	Hungary	Papp et al.	GA Generic Assays	ELISA	IgG	266	283	27	4	239	279	12
						IgA			9	1	257	282	
						either			27	5	239	278	
				Euroimmune	IIF	IgG			18	0	248	283	
						IgA			19	1	247	282	
						either			27	1	239	282	
[13]	2014	Czech Republic	Kohoutova et al.	GA Generic Assays	ELISA	IgG	86	70	27	5	59	65	6
						IgA			43	10	43	60	
						either NA			/	/	/	/	
[14]	2014	Netherlands	Gross et al.	GA Generic Assays	ELISA	IgG	38	136	7	5	31	131	11
						IgA			6	15	32	121	
						either NA			/	/	/	/	
[15]	2013	Germany	Komorowski et al.	In-house kit	IIF	IgG	96	50	5	0	91	50	10
						IgA			2	0	94	50	
						either NA			/	/	/	/	
[16]	2012	Serbia	Bonaci-Nikolic et al.	GA Generic Assays	ELISA	IgG	33	57	6	9	27	48	11
						IgA			8	9	25	48	
						either NA			/	/	/	/	

Supplementary Table 1 Characteristics of studies included in the meta-analysis of diagnostic performance of anti-glycoprotein 2 antibody in CD (continued)

Reference	Year	Region	Author	Manufacturer	Method	Ig subtype	Case	Control	TP	FP	FN	TN	QUADAS score
						IgG			59	16	166	284	
[17]	2012	United Kingdom	Pavlidis et al.	GA Generic Assays	ELISA	IgA NA	225	300	/	/	/	/	11
						either NA			/	/	/	/	
						IgG			48	14	121	313	
[18]	2012	Germany	Bogdanos et al.	GA Generic Assays	ELISA	IgA	169	327	22	5	147	322	11
						either			51	18	118	309	
						IgG			26	11	138	282	
[19]	2012	Belgium	Op De Beéck et al.	GA Generic Assays	ELISA	IgA	164	293	18	7	146	286	12
						either			34	18	130	275	
						IgG			13	4	57	46	
[20]	2011	United Kingdom	Pavlidis et al.	GA Generic Assays	ELISA	IgA	70	50	1	1	69	49	6
						either NA			/	/	/	/	
						IgG			53	12	125	250	
[21]	2011	Germany	Roggenbuck et al.	In-house kit	ELISA	IgA	178	262	24	4	154	258	11
						either			55	15	123	247	
						IgG			28	2	45	116	
[22]	2009	Germany	Roggenbuck et al.	In-house kit	IIF	IgA	73	118	18	0	55	118	11
						either NA			/	/	/	/	

Note: IIF: indirect immunofluorescence; ELISA: enzyme-linked immuno sorbent assay; NA: not applicable; TP: true positive; FP: false positive; TN: true negative; FN: false negative.
