# Supplementary material for: Diagnostic value of the antiglycoprotein-2 antibody for Crohn’s disease: a PRISMA-compliant systematic review and meta-analysis
Source: BMJ Open. 2017 Jun 9;7(6):e014843. doi: 10.1136/bmjopen-2016-014843 (PMC5734552; doi:10.1136/bmjopen-2016-014843)
Supplement: Author's manuscript [file bmjopen-2016-014843.draft_revisions.pdf]

BMJ Open

Diagnostic value of the anti-glycoprotein-2 antibody for Crohn's disease: A PRISMA-compliant systematic review and meta-analysis

Journal:	*BMJ Open*
Manuscript ID	bmjopen-2016-014843
Article Type:	Research
Date Submitted by the Author:	20-Oct-2016

Complete List of Authors:	Deng, Chuiwen; Peking Union Medical College Hospital, Chinese Academy of Medical Sciences and Peking Union Medical College, Rheumatology and Clinical Immunology<br>Li, Wenli<br>Li, Jing<br>Zhang, Shulan<br>Li, Yongzhe; Peking Union Medical College Hospital,Chinese Academy of Medical Sciences and Peking Union Medical College, Rheumatology and Clinical Immunology

**Primary Subject Heading**:	Gastroenterology and hepatology
Secondary Subject Heading:	Gastroenterology and hepatology
Keywords:	Inflammatory bowel disease < GASTROENTEROLOGY, Gastroenterology < INTERNAL MEDICINE, IMMUNOLOGY

SCHOLARONE™ Manuscripts

Diagnostic value of the anti-glycoprotein-2 antibody for Crohn's disease: A PRISMA-compliant systematic review and meta-analysis

Chuiwen Deng,[1¶] Wenli Li,[2¶] Jing Li,[3¶] Shulan Zhang,[1] Yongzhe Li,[1*]

[1]Department of Rheumatology and Clinical Immunology, Peking Union Medical College Hospital, Chinese Academy of Medical Sciences & Peking Union Medical College, Key Laboratory of Rheumatology and Clinical Immunology, Ministry of Education, Beijing, China.

[2]Department of Rheumatology, China-Japan Friendship Hospital, Beijing, China.

[3]Clinical laboratory, Peking university international hospital, Beijing, China.

[*]Correspondence author

Email address: yongzhelipumch@126.com (LYZ)

[¶]These authors are the co-first authors on this study.

Short title: anti-GP2 antibody in CD; a meta-analysis.

For peer review only - http://bmjopen.bmj.com/site/about/guidelines.xhtml

Abstract

Objectives: To perform a meta-analysis to evaluate the diagnostic value of the anti-glycoprotein 2 (GP2) antibody for Crohn's disease (CD).

Methods: Three databases—EMBASE, ISI Web of Knowledge and PubMed were systematically searched. There were 5,623 participants from several studies were included in this research. STATA 11.2 software and Meta-DiSc 1.4 were used to perform the meta-analysis.

Results: Seventeen studies were included in the meta-analysis. The pooled diagnostic sensitivity of anti-GP2 antibody ranged from 14%-24%. The specificity was 96%-98% and the area under the curve of the summary receiver operating characteristic was 0.68-0.72.

Conclusions: The anti-GP2 antibody is not a useful diagnostic biomarker for CD, as its sensitivity is extremely low.

Strengths and limitations of this study:

This is the first systematic review and meta-analysis that will evaluate the diagnostic value of the anti-glycoprotein 2 antibody for Crohn's disease. This research was prepared according to the Preferred Reporting Items for Systematic Review and Meta-Analysis.

Full-text screening, data extraction and analysis of included studies will be conducted by two reviewers independently.

Some relevant unpublished studies with negative findings that meet our inclusion criteria might be missed. Therefore, funnel plots will be used to detect possible publication bias to reach an objective conclusion.

Keywords: Anti-glycoprotein 2 antibody; Crohn's disease; Diagnostic performance; Meta-analysis

For peer review only - http://bmjopen.bmj.com/site/about/guidelines.xhtml

For peer review only

Introduction

Crohn's disease (CD) is a chronic inflammatory disorders of the gastrointestinal tract. Usually, the diagnosis of CD was based on current standard clinical, radiological, endoscopic, and histological criteria [1]. However, clinical symptom and radiological evidence are not specific. Meanwhile, endoscopic and histological tests are invasive operations that is difficult to be accept by patients. Precise diagnosis of CD and even differentiate CD from ulcerative colitis (UC) is urgently needed.

There is cumulative evidence from cross-sectional and longitudinal studies that support the value of serological markers applied in diagnosis, differential diagnosis, and prediction of disease course in CD [2]. The most widely used serological marker for CD is anti-Saccharomyces cerevisiae antibody. But its diagnostic performance in CD still could not achieve a high sensitivity and specificity for clinical need [3]. Nowadays, pancreatic autoantibody (PAB) has emerged as potentially diagnostic marker for CD [4]. Presence of PAB was identified first by indirect immunofluorescence technique [5]. Recently, the target antigens of PAB have been identified as the pancreatic major granule glycoprotein 2 (GP2) of the zymogen granule membrane and CUZD1 protein [4]. Now, anti-GP2 antibody can be detected by using enzyme-linked immuno sorbent assay in routine practice.

Over the past decade, numerous studies have evaluated the anti-GP2 antibody for its ability to accurately diagnose CD. However, inconsistent conclusions relating to the diagnostic performance of anti-GP2 antibody have been drawn [6-22]. The aim of the present study was to systematically review the literature to determine the diagnostic performance of anti-GP2 antibody in patients with CD.

Methods

For peer review only - http://bmjopen.bmj.com/site/about/guidelines.xhtml

Literature search

Studies were identified in the EMBASE, ISI Web of Knowledge and PubMed. To retrieve all relevant publications related to anti-GP2 antibody and CD, we searched for the follow terms: "anti-glycoprotein 2 antibody", "glycoprotein 2 autoantibodies" and "autoantibodies to glycoprotein 2", combined with "Crohn's disease" and "CD". No limits were placed on ethnicity or geographic region, and all documents were updated to Jun 2016. Additional relevant references cited in searched articles were also selected, if any. All analyses of this systemic review were based on previous published studies, thus no ethical approval and patient consent are required.

Eligibility criteria

Studies meeting the following criteria were eligible for inclusion: (1) those that assessed the diagnostic accuracy of anti-GP2 antibody for CD; (2) sufficient data reported to construct two-by-two tables; (3) no criteria for published language; and (4) studies based on animals or cell cultures, case reports and conference abstracts without subsequent publication in full text were excluded. In the case of overlapping studies, only the study with the largest sample size was included in our analysis.

Data extraction

Data were extracted from all selected studies by 2 independent investigators. Inter-researcher disagreements were resolved by consensus, or by a third investigator. The following data were collected from each selected study: first author's name; publication year; country in which the study was performed; and study results. Study quality was assessed using the Quality Assessment of Diagnostic Accuracy Studies (QUADAS) tool. Authors of the identified studies were contacted via e-mail if further study details were

For peer review only - http://bmjopen.bmj.com/site/about/guidelines.xhtml

needed.

Statistical analysis

Statistical analysis was performed using STATA 11.2 software (Stata Corporation, College Station, TX, USA) and Meta-DiSc 1.4 (Unit of Clinical Biostatistics, Ramon y Cajal Hospital, Madrid, Spain). Heterogeneity between studies was evaluated by Cochrane's Q-statistic as well as I^2-statistic. A P value > 0.10 in Q-statistic indicated lack of heterogeneity among studies. $I^2 < 25\%$ was considered low heterogeneity, 25–50% moderate, and > 50% a high degree of inconsistency. Finally, the overall or pooled sensitivity, specificity, positive likelihood ratio (LR+) and negative likelihood ratio (LR–), and their 95% CIs were obtained by a random-effects or a fixed-effects model in the presence ($P \leq 0.10$ or $I^2 > 50\%$) or absence ($P > 0.10$ and $I^2 \leq 50\%$) of heterogeneity, respectively. The area under the summary receiver operating characteristic (SROC) curves represented the overall performance of the detection method. A P value < 0.05 (two-sided) was considered significant. Evaluation of threshold effect and publication bias was also undertaken.

Results

Literature search

Electronic and manual searches yielded a total of 321 potentially eligible articles. A flow chart of screening articles for meta-analysis is illustrated in Fig 1. There were 221 articles excluded by screening the titles and abstracts. A further 19 full-tex articles were excluded for other reasons. Finally, a total of 17 eligible studies were included in the meta-analysis [6-22].

Study characteristics

For peer review only - http://bmjopen.bmj.com/site/about/guidelines.xhtml

The characteristics of the 17 studies are summarized in Supplementary Table 1. A total of 2439 CD patients and 3184 controls were involved in these studies. With regard to the geographic location of the studies, 7 were carried out in the Germany [7, 8, 11, 15, 18, 21, 22], 4 in United Kingdom [6, 10, 17, 20], and 1 in Serbia, Netherlands, Hungary, Czech Republic, Belgium and China [9, 12, 13, 14, 16, 19]. Assessment using QUADAS indicated that the studies were of high quality, with positive results for at least 6/14 items.

Meta-analysis of the diagnostic accuracy of anti-GP2 antibody (IgG) for CD

The diagnostic sensitivity of the anti-GP2 antibody (IgG) for CD ranged from 5–38%, and the reported specificity ranged from 84–100%. The diagnostic odds ratio was 7 (95% CI, 5-11; Q = 87.68, $P < 0.01$; $I^2 = 98\%$). The pooled sensitivity was 19% (95% CI, 14–25%; Q = 179.16, $P < 0.01$; $I^2 = 91\%$) and the pooled specificity was 97% (95% CI, 94–98%; Q = 75.97, $P < 0.01$; $I^2 = 78\%$). The LR+ and LR− were 6.1 (95% CI, 4.1–9.1), 0.84 (95% CI, 0.79–0.89), respectively. The area under the curve (AUC) of the SROC was 0.71. The sensitivity forest plots, specificity forest plots and SROC are shown in Figs 2A, 2B, and 2C, respectively.

Meta-analysis of the diagnostic accuracy of anti-GP2 antibody (IgA) for CD

The diagnostic sensitivity of the anti-GP2 antibody (IgA) for CD ranged from 1–50%, and the reported specificity ranged from 84–100%. The pooled sensitivity was 14% (95% CI, 9–20%; Q = 213.03, $P < 0.01$; $I^2 = 93\%$) and the pooled specificity was 98% (95% CI, 96–99%; Q = 156.06, $P < 0.01$; $I^2 = 90\%$). The LR+ and LR− were 6.3 (95% CI, 4.1–9.7), 0.88 (95% CI, 0.84–0.93), respectively. The AUC of the SROC was 0.68. The sensitivity forest plots, specificity forest plots and SROC are shown in Figs 3A, 3B, and 3C, respectively.

For peer review only - http://bmjopen.bmj.com/site/about/guidelines.xhtml

Meta-analysis of the diagnostic accuracy of anti-GP2 antibody (either IgG or IgA) for CD

The diagnostic sensitivity of the anti-GP2 antibody (IgA) for CD ranged from 10–54%, and the reported specificity ranged from 84–100%. The pooled sensitivity was 24% (95% CI, 18–32%; Q = 103.59, $P < 0.01$; $I^2 = 90\%$) and the pooled specificity was 96% (95% CI, 93–97%; Q = 55.87, $P < 0.01$; $I^2 = 82\%$). The LR+ and LR− were 5.4 (95% CI, 4.1-7.2), 0.79 (95% CI, 0.74–0.85), respectively. The AUC of the SROC was 0.72. The sensitivity forest plots, specificity forest plots and SROC are shown in Figs 4A, 4B, and 4C, respectively.

Multiple regression analysis and exploration of threshold effect

Meta-regression analysis was conducted to explore possible sources of heterogeneity across the 17 included studies. The following co-variates were evaluated as predictor variables; the method of autoantibody detection, the manufacturer of detection kits, the region of the study performed and the QUADAS scores. Results indicated that the method of autoantibody detection led to the heterogeneity of the diagnostic specificity of anti-GP2 antibody (IgG) in CD, and no other sources of heterogeneity was found.

A Spearman's rank correlation was performed to confirm the threshold effect; indication of no threshold effect was found (Spearman's correlation coefficient and P-value for anti-GP2 antibody (IgG), anti-GP2 antibody (IgA), and anti-GP2 antibody (either IgG or IgA) were 0.525 and 0.025, 0.622 and 0.008, 0.761 and 0.007, respectively.

Publication bias

The presence of a statistically significant slope coefficient ($P < 0.05$) was considered to indicate possible bias. We conducted funnel plots that represented a somewhat symmetric curve (Fig 5). The P value of the

For peer review only - http://bmjopen.bmj.com/site/about/guidelines.xhtml

slope coefficient for anti-GP2 antibody (IgG), anti-GP2 antibody (IgA), and anti-GP2 antibody (either IgG or IgA) were calculated to be 0.11, 0.10 and 0.22, respectively, indicating that no publication bias was observed in the included studies.

Discussion

The diagnosis of CD mainly relies on a triad of clinical characteristics: radiological features, endoscopic and histological assessment. However, the common clinical manifestations are not specific to CD. Furthermore, radiological, endoscopic and histological assessments are invasive that not easy for patients and clinicians to accept. To date, anti-GP2 antibody is considering as an emerging important factors for diagnosing of CD. To our knowledge, this study is the first meta-analysis to provide precise and controlled data on the diagnostic performance of the anti-GP2 antibody in CD.

There were 17 eligible studies of high quality included in this meta-analysis (Supplementary Table 1). To explore the diagnosis performance of anti-GP2 antibody IgG subtype, IgA subtype, and either IgG or IgA subtype, we calculated the statistics of them, respectively. In anti-GP2 antibody IgG subtype, the pooled specificity was high (97%), in comparison with a relatively low sensitivity (19%), which revealed that the diagnostic performance of the anti-GP2 antibody IgG subtype could identify controls better than CD. The AUC (0.71) of the SROC curve implies that the anti-GP2 antibody IgG subtype showed unsatisfied diagnostic performance for CD. Taken together, these results indicate that the anti-GP2 antibody IgG subtype had a low level of overall diagnostic accuracy for CD. Comparing with IgG subtype, anti-GP2 antibody IgA subtype show higher specificity (98%), but lower sensitivity (14%). In addition, the AUC of anti-GP2 antibody IgA subtype related SROC curve was 0.68, which also implies an unsatisfied diagnostic

For peer review only

For peer review only - http://bmjopen.bmj.com/site/about/guidelines.xhtml

performance for CD. The diagnosis accuracy was more satisfied when considering both IgG subtype and IgA subtype in CD diagnosis (pooled sensitivity, 24%; pooled specificity, 96%; the AUC of SROC, 0.72). Since there is a so high difference in sensitivity and specificity of the above immune subtypes, the diagnostic power as expressed by AUC loses any significance, and makes unlikely any use of this autoantibody in clinical practice, as sensitivity is extremely low.

Preliminary analysis showed that detecting both the IgG and IgA subtype of anti-GP2 antibody owned a better diagnostic value for CD than detecting IgA or IgG subtype along. Heterogeneity has been found in both the pooled sensitivity and specificity of the anti-GP2 antibody when detecting both the IgG and IgA subtype. Then, we conducted a meta-regression analysis for anti-GP2 antibody. However, no significance was found among the method of autoantibody detection, the manufacturer of detection kits, the region of the study performed and the QUADAS scores when detecting both the IgG and IgA subtype. Interestingly, the method of autoantibody detection was confirmed to be the source of the heterogeneity of the diagnostic specificity of anti-GP2 antibody (IgG) in CD. Publication bias and Spearman's rank correlation also has been performed. No threshold effect and publication bias was found in this meta-analysis.

The limitations in this meta-analysis need to be noted, that we did not compare the diagnosis accuracy of anti-GP2 antibody with anti-Saccharomyces cerevisiae antibody for the lack of researches.

To sum up, the anti-GP2 antibody is not a useful diagnostic biomarker for CD, as its sensitivity is extremely low.

Author contribution: LYZ designed the research, DCW, LWL and LJ collected and analysis the data, DCW wrote the paper, LYZ and ZSL help optimize the research and proofread the paper.

For peer review only - http://bmjopen.bmj.com/site/about/guidelines.xhtml

Funding statement: This work was supported by funding from the Research Special Fund for Public Welfare Industry of Health (201202004), and the National Natural Science Foundation of China Grants (81302591, 81172857, 81373188), the Chinese National High Technology Research and Development Program, Ministry of Science and Technology Grants (2011AA02A113), and the National Science Technology Pillar Program in the 12nd Five-year Plan (2014BAI07B00).

Competing interest: The authors declare that no competing interests exist.

Data sharing statement: All raw data are supplied in the supplementary table 1. All recorded information from the data extraction process, not included in the systematic review article, will be available on request.

Reference

[1] Lennard-Jones JE. Classification of inflammatory bowel disease. Scand J Gastroenterol Suppl. 1989;170:2-6.

[2] Laass MW, Roggenbuck D, Conrad K. Diagnosis and classification of Crohn's disease. Autoimmun Rev. 2014;13:467-71.

[3] Bossuyt X. Serologic markers in inflammatory bowel disease. Clin Chem. 2006;52:171-81.

[4] Roggenbuck D, Reinhold D, Wex T, Goihl A, von Arnim U, Malfertheiner P, et al. Autoantibodies to GP2, the major zymogen granule membrane glycoprotein, are new markers in Crohn's disease. Clin Chim Acta. 2011;412:718-724.

[5] Stöcker W, Otte M, Ulrich S, Normann D, Finkbeiner H, Stöcker K, et al. Autoimmunity to pancreatic juice in Crohn's disease. Results of an autoantibody screening in patients with chronic inflammatory bowel disease. Scand J Gastroenterol Suppl. 1987;139:41-52.

For peer review only - http://bmjopen.bmj.com/site/about/guidelines.xhtml

[6] Pavlidis P, Komorowski L, Teegen B, Liaskos C, Koutsoumpas AL, Smyk DS, et al. Diagnostic and clinical significance of Crohn's disease-specific pancreatic anti-GP2 and anti-CUZD1 antibodies. Clin Chem Lab Med. 2016;54:249-256.

[7] Michaels MA, Jendrek ST, Korf T, Nitzsche T, Teegen B, Komorowski L, et al. Pancreatic Autoantibodies Against CUZD1 and GP2 Are Associated with Distinct Clinical Phenotypes of Crohn's Disease. Inflamm Bowel Dis. 2015;21:2864-2872.

[8] Roggenbuck D, Vermeire S, Hoffman I, Reinhold D, Schierack P, Goihl A, et al. Evidence of Crohn's disease-related anti-glycoprotein 2 antibodies in patients with celiac disease. Clin Chem Lab Med. 2015;53:1349-1357.

[9] Zhang S, Wu Z, Luo J, Ding X, Hu C, Li P, et al. Diagnostic Potential of Zymogen Granule Glycoprotein 2 Antibodies as Serologic Biomarkers in Chinese Patients With Crohn Disease. Medicine (Baltimore). 2015;94:e1654.

[10] Pavlidis P, Shums Z, Koutsoumpas AL, Milo J, Papp M, Umemura T, et al. Diagnostic and clinical significance of Crohn's disease-specific anti-MZGP2 pancreatic antibodies by a novel ELISA. Clin Chim Acta. 2015;441:176-181.

[11] Laass MW, Röber N, Range U, Noß L, Roggenbuck D, Conrad K. Loss and Gain of Tolerance to Pancreatic Glycoprotein 2 in Celiac Disease. PLoS One. 2015;10:e0128104.

[12] Papp M, Sipeki N, Tornai T, Altorjay I, Norman GL, Shums Z, et al. Rediscovery of the Anti-Pancreatic Antibodies and Evaluation of their Prognostic Value in a Prospective Clinical Cohort of Crohn's Patients: The Importance of Specific Target Antigens [GP2 and CUZD1]. J Crohns Colitis.

For peer review only - http://bmjopen.bmj.com/site/about/guidelines.xhtml

2015;9:659-668.

[13] Kohoutova D, Drahosova M, Moravkova P, Rejchrt S, Bures J. Anti-Outer membrane protein C and anti-glycoprotein 2 antibodies in inflammatory bowel disease and their association with complicated forms of Crohn's disease. BMC Gastroenterol. 2014;14:190.

[14] Gross S, Bakker SF, van Bodegraven AA, van Hoogstraten IM, Gelderman KA, Bouma G, et al. Increased IgA glycoprotein-2 specific antibody titres in refractory celiac disease. J Gastrointestin Liver Dis. 2014;23:127-133.

[15] Komorowski L, Teegen B, Probst C, Aulinger-Stöcker K, Sina C, Fellermann K, et al. Autoantibodies against exocrine pancreas in Crohn's disease are directed against two antigens: the glycoproteins CUZD1 and GP2. J Crohns Colitis. 2013;7:780-790.

[16] Bonaci-Nikolic B, Spuran M, Andrejevic S, Nikolic M. Autoantibodies to GP2, the major zymogen granule membrane glycoprotein, in patients with gluten-sensitive enteropathy: a possible serological trap. Clin Chim Acta. 2012;413:822-823.

[17] Pavlidis P, Romanidou O, Roggenbuck D, Mytilinaiou MG, Al-Sultan F, Liaskos C, et al. Ileal inflammation may trigger the development of GP2-specific pancreatic autoantibodies in patients with Crohn's disease. Clin Dev Immunol. 2012;2012:640835.

[18] Bogdanos DP, Roggenbuck D, Reinhold D, Wex T, Pavlidis P, von Arnim U, et al. Pancreatic-specific autoantibodies to glycoprotein 2 mirror disease location and behaviour in younger patients with Crohn's disease. BMC Gastroenterol. 2012;12:102.

[19] Op De Beéck K, Vermeire S, Rutgeerts P, Bossuyt X. Antibodies to GP2, the major zymogen granule

For peer review only - http://bmjopen.bmj.com/site/about/guidelines.xhtml

membrane glycoprotein, in inflammatory bowel diseases. Gut. 2012;61:162-4; author reply 164-165.

[20] Pavlidis P, Forbes A, Bogdanos DP. Antibodies to glycoprotein 2 (GP2) in patients with inflammatory bowel diseases from UK. Clin Chim Acta. 2011;412:1163-1164.

[21] Roggenbuck D, Reinhold D, Wex T, Goihl A, von Arnim U, Malfertheiner P, et al. Autoantibodies to GP2, the major zymogen granule membrane glycoprotein, are new markers in Crohn's disease. Clin Chim Acta. 2011;412:718-724.

[22] Roggenbuck D, Hausdorf G, Martinez-Gamboa L, Reinhold D, Büttner T, Jungblut PR, et al. Identification of GP2, the major zymogen granule membrane glycoprotein, as the autoantigen of pancreatic antibodies in Crohn's disease. Gut. 2009;58:1620-1628.

Figure legend

Fig 1. Flow chart of studies included in the meta-analysis.

Fig 2. The forest plots and the summary receiver operating characteristic curves of anti-glycoprotein 2 antibody (IgG) for CD. A: Sensitivity forest plot; B: Specificity forest plot; C: sROC.

Fig 3. The forest plots and the summary receiver operating characteristic curves of anti-glycoprotein 2 antibody (IgA) for CD. A: Sensitivity forest plot; B: Specificity forest plot; C: sROC.

Fig 4. The forest plots and the summary receiver operating characteristic curves of anti-glycoprotein 2 antibody (either IgG or IgA) for CD. A: Sensitivity forest plot; B: Specificity forest plot; C: sROC.

Fig 5. Funnel plot of the publication bias for the anti-glycoprotein 2 antibody (IgG and IgA either)

For peer review only

For peer review only - http://bmjopen.bmj.com/site/about/guidelines.xhtml

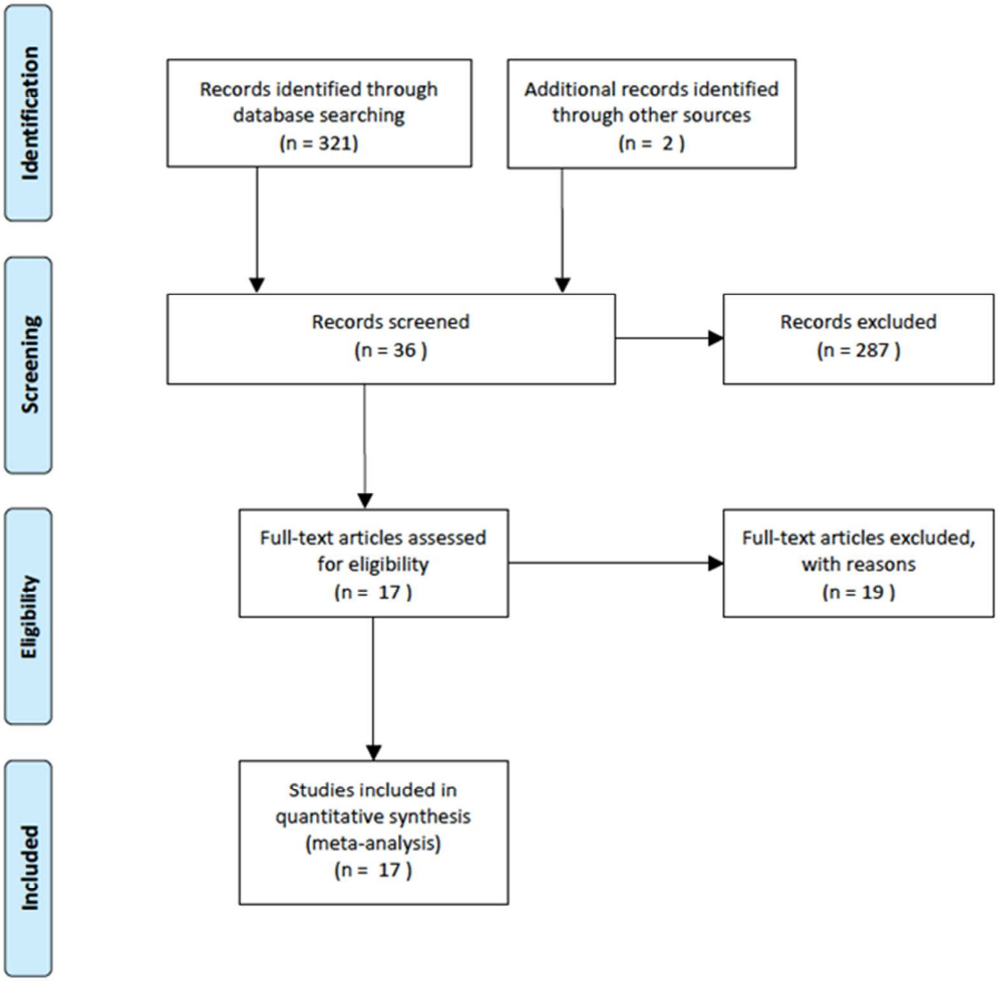

193x190mm (300 x 300 DPI)

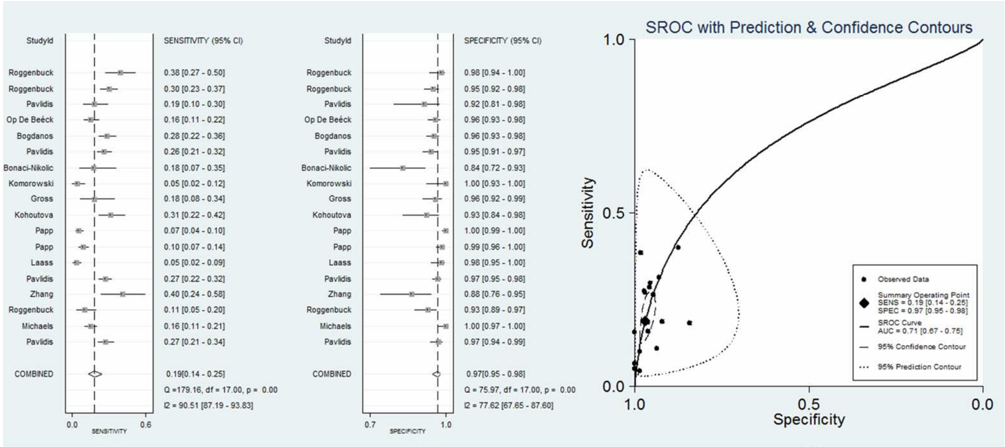

(A) (B) (C)

307x145mm (300 x 300 DPI)

For peer review only

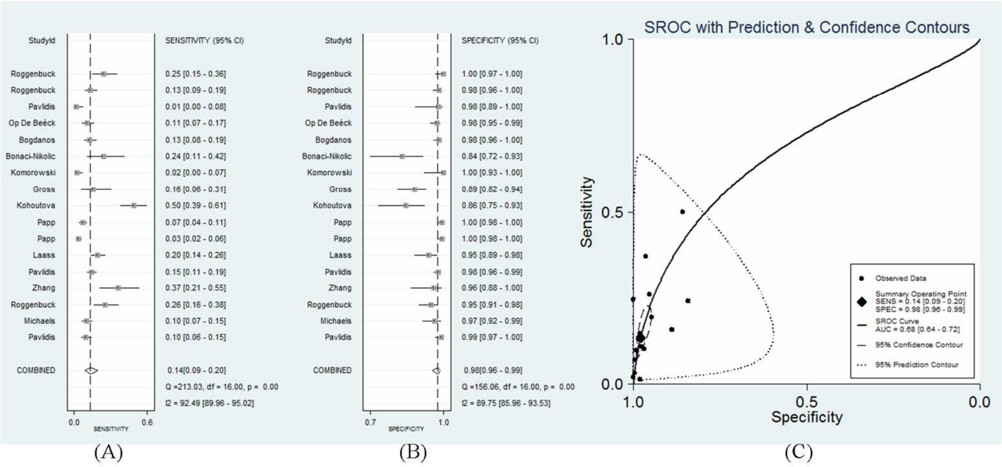

310x145mm (300 x 300 DPI)

er review only

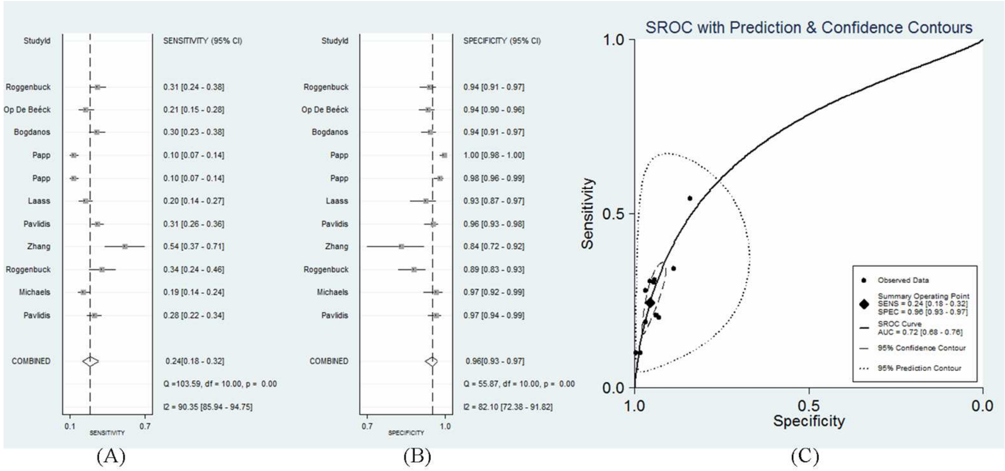

(A) (B) (C)

308x145mm (300 x 300 DPI)

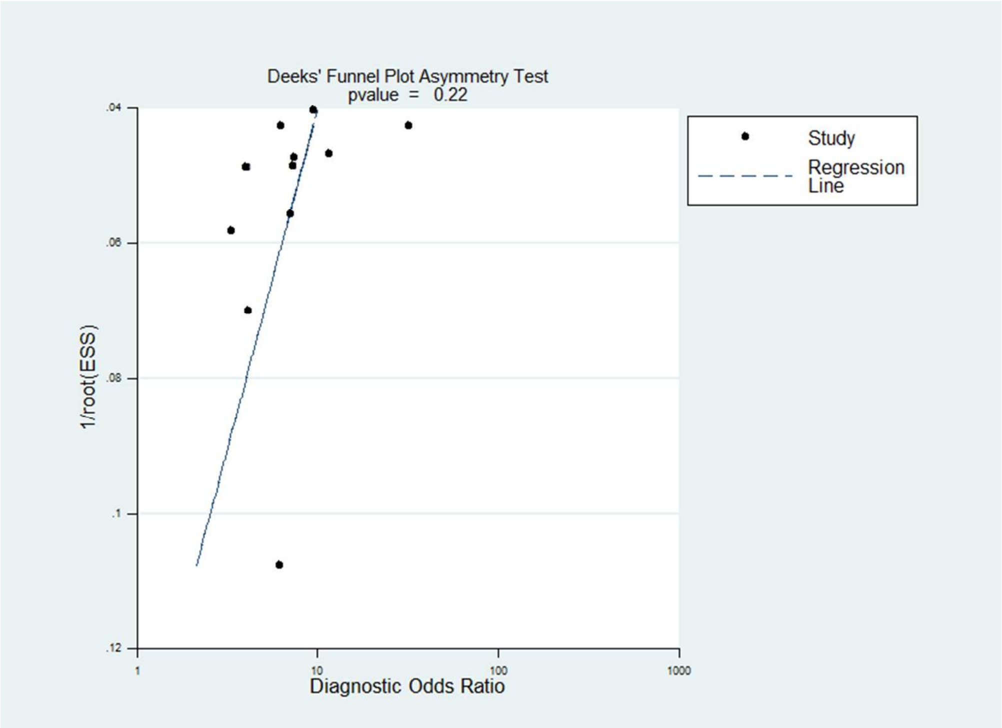

292x212mm (300 x 300 DPI)

For peer review only

Supplementary Table 1 Characteristics of studies included in the meta-analysis of diagnostic performance of anti-granule protein 2 antibody in CD

Reference	Year	Region	Author	Manufacturer	Method	Ig subtype	Case	Control	TP	FP	FN	TN	QUADAS score
						IgG			58	7	154	242	
[6]	2016	United Kingdom	Pavlidis et al.	In-house kit	IIF	IgA	212	249	21	2	191	247	12
						either			59	8	153	241	
						IgG			35	0	189	126	
[7]	2015	Germany	Michaels et al.	Euroimmune	IIF	IgA	224	126	23	4	201	122	12
						either			42	4	182	122	
						IgG			8	11	65	158	
[8]	2015	Germany	Roggenbuck et al.	GA Generic Assays	ELISA	IgA	73	169	19	8	54	161	11
						either			25	19	48	150	
						IgG			14	7	21	49	
[9]	2015	China	Zhang et al.	GA Generic Assays	ELISA	IgA	35	56	13	2	22	54	11
						either			19	9	16	47	
						IgG	323	509	87	15	236	494	
[10]	2015	United Kingdom	Pavlidis et al.	Inova Diagnostics	ELISA	IgA			48	10	275	499	12
						either	323	294	99	13	224	281	
						IgG			8	2	166	127	
[11]	2015	Germany	Laass et al.	GA Generic Assays	ELISA	IgA	174	129	34	7	140	122	10
						either			35	9	139	120	
[12]	2015	Hungary	Papp et al.	GA Generic Assays	ELISA	IgG	266	283	27	4	239	279	12
						IgA			9	1	257	282	

For peer review only - http://bmjopen.bmj.com/site/about/guidelines.xhtml

						either			27	5	239	278	
						IgG			18	0	248	283	
				Euroimmune	IIF	IgA			19	1	247	282	
						either			27	1	239	282	
						IgG			27	5	59	65	
[13]	2014	Czech Republic	Kohoutova et al.	GA Generic Assays	ELISA	IgA	86	70	43	10	43	60	6
						either NA			/	/	/	/	
						IgG			7	5	31	131	
[14]	2014	Netherlands	Gross et al.	GA Generic Assays	ELISA	IgA	38	136	6	15	32	121	11
						either NA			/	/	/	/	
						IgG			5	0	91	50	
[15]	2013	Germany	Komorowski et al.	In-house kit	IIF	IgA	96	50	2	0	94	50	10
						either NA			/	/	/	/	
						IgG			6	9	27	48	
[16]	2012	Serbia	Bonaci-Nikolic et al.	GA Generic Assays	ELISA	IgA	33	57	8	9	25	48	11
						either NA			/	/	/	/	
						IgG			59	16	166	284	
[17]	2012	United Kingdom	Pavlidis et al.	GA Generic Assays	ELISA	IgA NA	225	300	/	/	/	/	11
						either NA			/	/	/	/	
						IgG			48	14	121	313	
[18]	2012	Germany	Bogdanos et al.	GA Generic Assays	ELISA	IgA	169	327	22	5	147	322	11
						either			51	18	118	309	
						IgG			26	11	138	282	
[19]	2012	Belgium	Op De Beéck et al.	GA Generic Assays	ELISA	IgA	164	293	18	7	146	286	12
						either			34	18	130	275	

For peer review only

						IgG			13	4	57	46	
[20]	2011	United Kingdom	Pavlidis et al.	GA Generic Assays	ELISA	IgA	70	50	1	1	69	49	6
						either NA			/	/	/	/	
						IgG			53	12	125	250	
[21]	2011	Germany	Roggenbuck et al.	In-house kit	ELISA	IgA	178	262	24	4	154	258	11
						either			55	15	123	247	
						IgG			28	2	45	116	
[22]	2009	Germany	Roggenbuck et al.	In-house kit	IIF	IgA	73	118	18	0	55	118	11
						either NA			/	/	/	/	

Note: IIF: indirect immunofluorescence; ELISA: enzyme-linked immuno sorbent assay; NA: not applicable; TP: true positive; FP: false positive; TN: true negative; FN: false negative.

For peer review only

PRISMA 2009 Checklist

Section/Topic	#	Checklist Item	Reported on Page #
**TITLE**			
Title	1	Identify the report as a systematic review, meta-analysis, or both.	1
**ABSTRACT**			
Structured summary	2	Provide a structured summary including, as applicable: background; objectives; data sources; study eligibility criteria, participants, and interventions; study appraisal and synthesis methods; results; limitations; conclusions and implications of key findings; systematic review registration number.	2
**INTRODUCTION**			
Rationale	3	Describe the rationale for the review in the context of what is already known.	3
Objectives	4	Provide an explicit statement of questions being addressed with reference to participants, interventions, comparisons, outcomes, and study design (PICOS).	3
**METHODS**			
Protocol and registration	5	Indicate if a review protocol exists, if and where it can be accessed (e.g., Web address), and, if available, provide registration information including registration number.	Not applicable
Eligibility criteria	6	Specify study characteristics (e.g., PICOS, length of follow-up) and report characteristics (e.g., years considered, language, publication status) used as criteria for eligibility, giving rationale.	4
Information sources	7	Describe all information sources (e.g., databases with dates of coverage, contact with study authors to identify additional studies) in the search and date last searched.	4
Search	8	Present full electronic search strategy for at least one database, including any limits used, such that it could be repeated.	4
Study selection	9	State the process for selecting studies (i.e., screening, eligibility, included in systematic review, and, if applicable, included in the meta-analysis).	4
Data collection process	10	Describe method of data extraction from reports (e.g., piloted forms, independently, in duplicate) and any processes for obtaining and confirming data from investigators.	4
Data items	11	List and define all variables for which data were sought (e.g., PICOS, funding sources) and any assumptions and simplifications made.	4
Risk of bias in individual studies	12	Describe methods used for assessing risk of bias of individual studies (including specification of whether this was done at the study or outcome level), and how this information is to be used in any data synthesis.	Not applicable
Summary measures	13	State the principal summary measures (e.g., risk ratio, difference in means).	4
Synthesis of results	14	Describe the methods of handling data and combining results of studies, if done, including measures of consistency (e.g., I^2 for each meta-analysis.	5

For peer review only - http://bmjopen.bmj.com/site/about/guidelines.xhtml

PRISMA 2009 Checklist

Section/Topic	#	Checklist Item	Reported on Page #
Risk of bias across studies	15	Specify any assessment of risk of bias that may affect the cumulative evidence (e.g., publication bias, selective reporting within studies).	5
Additional analyses	16	Describe methods of additional analyses (e.g., sensitivity or subgroup analyses, meta-regression), if done, indicating which were pre-specified.	5
**RESULTS**			
Study selection	17	Give numbers of studies screened, assessed for eligibility, and included in the review, with reasons for exclusions at each stage, ideally with a flow diagram.	5
Study characteristics	18	For each study, present characteristics for which data were extracted (e.g., study size, PICOS, follow-up period) and provide the citations.	6
Risk of bias within studies	19	Present data on risk of bias of each study and, if available, any outcome level assessment (see item 12).	Not applicable
Results of individual studies	20	For all outcomes considered (benefits or harms), present, for each study: (a) simple summary data for each intervention group (b) effect estimates and confidence intervals, ideally with a forest plot.	5-8
Synthesis of results	21	Present the main results of the review. If meta-analyses done, include for each, confidence intervals and measures of consistency.	6-7
Risk of bias across studies	22	Present results of any assessment of risk of bias across studies (see Item 15).	Not applicable
Additional analysis	23	Give results of additional analyses, if done (e.g., sensitivity or subgroup analyses, meta-regression [see Item 16]).	7-8
**DISCUSSION**			
Summary of evidence	24	Summarize the main findings including the strength of evidence for each main outcome; consider their relevance to key groups (e.g., healthcare providers, users, and policy makers).	8
Limitations	25	Discuss limitations at study and outcome level (e.g., risk of bias), and at review-level (e.g., incomplete retrieval of identified research, reporting bias).	9
Conclusions	26	Provide a general interpretation of the results in the context of other evidence, and implications for future research.	9
**FUNDING**			
Funding	27	Describe sources of funding for the systematic review and other support (e.g., supply of data); role of funders for the systematic review.	10

From: Moher D, Liberati A, Tetzlaff J, Altman DG, The PRISMA Group (2009). Preferred Reporting Items for Systematic Reviews and Meta-Analyses: The PRISMA Statement. PLoS Med 6(6): e1000097. doi:10.1371/journal.pmed1000097

For peer review only - http://bmjopen.bmj.com/site/about/guidelines.xhtml

PRISMA 2009 Checklist

For more information, visit: www.prisma-statement.org.

For peer review only

For peer review only - http://bmjopen.bmj.com/site/about/guidelines.xhtml

BMJ Open

Diagnostic value of the anti-glycoprotein-2 antibody for Crohn's disease: A PRISMA-compliant systematic review and meta-analysis

Journal:	*BMJ Open*
Manuscript ID	bmjopen-2016-014843.R1
Article Type:	Research
Date Submitted by the Author:	20-Feb-2017
Complete List of Authors:	Deng, Chuiwen; Peking Union Medical College Hospital, Chinese Academy of Medical Sciences and Peking Union Medical College, Rheumatology and Clinical Immunology<br>Li, Wenli<br>Li, Jing<br>Zhang, Shulan<br>Li, Yongzhe; Peking Union Medical College Hospital,Chinese Academy of Medical Sciences and Peking Union Medical College, Rheumatology and Clinical Immunology
**Primary Subject Heading**:	Gastroenterology and hepatology
Secondary Subject Heading:	Gastroenterology and hepatology
Keywords:	Inflammatory bowel disease < GASTROENTEROLOGY, Gastroenterology < INTERNAL MEDICINE, IMMUNOLOGY

SCHOLARONE™
Manuscripts

For peer review only

Diagnostic value of the anti-glycoprotein-2 antibody for Crohn's disease: A PRISMA-compliant systematic review and meta-analysis

Chuiwen Deng,[1¶] Wenli Li,[2¶] Jing Li,[3¶] Shulan Zhang,[1] Yongzhe Li,[1*]

[1]Department of Rheumatology and Clinical Immunology, Peking Union Medical College Hospital, Chinese Academy of Medical Sciences & Peking Union Medical College, Key Laboratory of Rheumatology and Clinical Immunology, Ministry of Education, Beijing, China.

[2]Department of Rheumatology, China-Japan Friendship Hospital, Beijing, China.

[3]Clinical laboratory, Peking university international hospital, Beijing, China.

[*]Correspondence author

Email address: yongzhelipumch@126.com (LYZ)

[¶]These authors are the co-first authors on this study.

Short title: Anti-GP2 antibody in CD, a meta-analysis

For peer review only

For peer review only - http://bmjopen.bmj.com/site/about/guidelines.xhtml

Abstract

Objectives: To perform a meta-analysis to evaluate the diagnostic performance of the anti-glycoprotein-2 (GP2) antibody for Crohn's disease (CD).

Methods: Three databases (EMBASE, ISI Web of Knowledge, and PubMed) were systematically searched. There were 17 eligible studies included in the meta-analysis. A total of 2,439 CD patients and 3,184 controls were involved in these studies. STATA 11.2 and Meta-DiSc 1.4 were used to perform the meta-analysis.

Results: The pooled diagnostic sensitivity of the anti-GP2 antibody ranged from 14 to 24%. The specificity was 96–98%, and the area under the summary receiver operating characteristic curve was 0.68–0.72.

Conclusions: The anti-GP2 antibody is a specific biomarker for CD, and further exploration of its prevalence among different clinical phenotypes of CD will provide a better understanding of its diagnostic performance.

For peer review only

Article summary

Strengths and limitations of this study:

1. This meta-analysis was prepared according to the Preferred Reporting Items for Systematic Review and Meta-Analysis.

2. The methods that were applied in this meta-analysis included evaluations of the threshold effect, pooled statistics, operating characteristic curve, publication bias, and other sources of heterogeneity.

3. The full-text screening, data extraction, and analysis of the included studies were conducted by two reviewers independently.

For peer review only - http://bmjopen.bmj.com/site/about/guidelines.xhtml

4. This is the first systematic review and meta-analysis to evaluate the diagnostic performance of the anti-glycoprotein-2 antibody for Crohn's disease.

Keywords: Anti-glycoprotein 2 antibody; Crohn's disease; Diagnostic performance; Meta-analysis

For peer review only

Introduction

Crohn's disease (CD) is a chronic inflammatory disorder of the gastrointestinal tract. Usually, the diagnosis of CD is based on current standard clinical, radiological, endoscopic, and histological criteria [1]. However, clinical symptoms and radiological evidence are not specific, while endoscopic and histological tests are invasive procedures that are not always accepted by patients. A precise method for diagnosing CD and differentiating it from ulcerative colitis (UC) is urgently needed.

There is cumulative evidence from cross-sectional and longitudinal studies to support the value of serological markers in the diagnosis, differential diagnosis, and prediction of disease course in CD [2]. The most widely used serological marker for CD is the anti-*Saccharomyces cerevisiae* antibody. However, its diagnostic performance in CD has not achieved sufficiently high sensitivity or specificity for clinical needs [3]. Recently, pancreatic autoantibody (PAB) has emerged as a potential diagnostic marker for CD [4]. The presence of PAB was first identified by indirect immunofluorescence [5]. Recently, the target antigens of PAB have been identified as the pancreatic major granule glycoprotein 2 (GP2) of the zymogen granule membrane and the CUZD1 protein [4]. The anti-GP2 antibody can be detected using an enzyme-linked immunosorbent assay in routine practice.

Over the past decade, numerous studies have evaluated the anti-GP2 antibody for its ability to accurately diagnose CD. However, inconsistent conclusions relating to the diagnostic performance of anti-GP2 antibody have been drawn [6-22]. The reported diagnostic sensitivity of anti-GP2 antibody (IgG) ranges from 5 to 40%, and the reported diagnostic specificity ranges from 84 to 100%. The IgA subtype of the anti-GP2 antibody is also considered to be potentially valuable in CD diagnosis, but the reported

For peer review only - http://bmjopen.bmj.com/site/about/guidelines.xhtml

sensitivities (1–50%) and specificities (84–100%) also show high variation. In addition, some researchers have proposed that measurement of either the IgG or IgA subtype of the anti-GP2 antibody would improve its diagnostic value of CD, while others have disagreed. Lastly, methodological discrepancies, including the subtypes of CD tested, the method of autoantibody detection, and the manufacturer of the detection kits, might influence the diagnostic value of the anti-GP2 antibody, and these should be investigated.

In order to verify the diagnostic performance of the anti-GP2 antibody in patients with CD and to determine the factors that influence the results of anti-GP2 antibody testing, we performed the present systematic review and meta-analysis

Methods

Literature search

Studies were identified in EMBASE, ISI Web of Knowledge, and PubMed databases. To retrieve all relevant publications related to anti-GP2 antibody and CD, we searched for the follow terms: “anti-glycoprotein 2 antibody”, “glycoprotein 2 autoantibodies”, and “autoantibodies to glycoprotein 2”, combined with “Crohn’s disease” and “CD”. No limits were placed on ethnicity or geographic region, and all documents were included up to June 2016. The specific PubMed search algorithm is provided in Supplementary File 1. Additional relevant references cited in searched articles were also selected, if any. All analyses in this systemic review were based on previously published studies, and thus no ethical approval or patient consent was required.

Eligibility criteria

The following criteria were used to determine eligibility for inclusion: (1) studies that assessed the

For peer review only - http://bmjopen.bmj.com/site/about/guidelines.xhtml

For peer review only

diagnostic accuracy of the anti-GP2 antibody for CD were included; (2) studies with sufficient data to construct two-by-two tables were included; (3) all published languages were included; and (4) studies based on animals or cell cultures or case reports without subsequent publication in full text were excluded. In the case of overlapping studies, only the study with the largest sample size was included in our analysis.

Data extraction

Data were extracted from all selected studies by two independent investigators. Inter-researcher disagreements were resolved by consensus or by a third investigator. The following data were collected from each selected study: first author's name; publication year; country in which the study was performed; and study results. Study quality was assessed using the Quality Assessment of Diagnostic Accuracy Studies (QUADAS) tool. Authors of the identified studies were contacted via e-mail if further study details were needed.

Statistical analysis

Statistical analysis was performed using STATA 11.2 (Stata Corporation, College Station, TX, USA) and Meta-DiSc 1.4 (Unit of Clinical Biostatistics, Ramon y Cajal Hospital, Madrid, Spain). Potential important differences in the results of the individual studies are frequently referred to as heterogeneity. In this meta-analysis, the heterogeneity among studies was evaluated by Cochrane's Q-statistic as well as by the I^2-statistic. A Q-statistic P-value > 0.10 indicated lack of heterogeneity among studies. $I^2 < 25\%$ was considered low heterogeneity, 25–50% moderate, and > 50% indicated a high degree of heterogeneity. Finally, the overall or pooled sensitivity, specificity, positive likelihood ratio (LR+), negative likelihood ratio (LR–), and their 95% confidence intervals (Cis) were obtained by a random-effects or a fixed-effects

For peer review only - http://bmjopen.bmj.com/site/about/guidelines.xhtml

model in the presence (P ≤ 0.10 or I^2 > 50%) or absence (P > 0.10 and I^2 ≤ 50%) of heterogeneity, respectively. The area under the summary receiver operating characteristic (SROC) curve represented the overall performance of the detection method. A P-value < 0.05 (two-sided) was considered significant. Threshold effects occur when different cut-off values are applied in different studies, leading to heterogeneity of the results. Besides assessing threshold effects from the SROCs and forest plots, we also performed a Spearman's rank correlation to test for threshold effects. Evaluations of publication bias was also undertaken.

Results

Literature search

Electronic and manual searches yielded a total of 323 potentially eligible articles. A flow chart of the screening of articles for meta-analysis is illustrated in Fig 1. There were 287 articles that were excluded based on screening of the titles and abstracts. A further 19 full-text articles were excluded as they were not related to our subject. Finally, a total of 17 eligible studies were included in the meta-analysis [6-22].

Study characteristics

The characteristics of the 17 studies are summarized in Supplementary Table 1. A total of 2,439 CD patients and 3,184 controls were involved in these studies. With regard to the geographic location of the studies, seven were carried out in Germany [7, 8, 11, 15, 18, 21, 22], four in the United Kingdom [6, 10, 17, 20], and one each in Serbia, the Netherlands, Hungary, the Czech Republic, Belgium, and China [9, 12, 13, 14, 16, 19]. Assessment using QUADAS indicated that the studies were of high quality, with positive results for at least 6/14 items.

For peer review only - http://bmjopen.bmj.com/site/about/guidelines.xhtml

Meta-analysis of the diagnostic accuracy of the anti-GP2 antibody (IgG) for CD

A total of 2,439 CD patients and 3,184 controls were involved in this part of the meta-analysis. The diagnostic sensitivity of the anti-GP2 antibody (IgG) for CD ranged from 5–38%, and the reported specificity ranged from 84–100% [6-22]. The diagnostic odds ratio was 7 (95% CI, 5–11; Q = 87.68, P < 0.01; I^2 = 98%). The pooled sensitivity was 19% (95% CI, 14–25%; Q = 179.16, P < 0.01; I^2 = 91%), and the pooled specificity was 97% (95% CI, 94–98%; Q = 75.97, P < 0.01; I^2 = 78%). The LR+ and LR− were 6.1 (95% CI, 4.1–9.1) and 0.84 (95% CI, 0.79–0.89), respectively. The area under the curve (AUC) of the SROC was 0.71 (95% CI, 0.67–0.75). The sensitivity forest plots, specificity forest plots, and SROC are shown in Figs 2A, 2B, and 2C, respectively.

Meta-analysis of the diagnostic accuracy of the anti-GP2 antibody (IgA) for CD

A total of 2,214 CD patients and 2,884 controls were involved in this part of the meta-analysis. The diagnostic sensitivity of the anti-GP2 antibody (IgA) for CD ranged from 1–50%, and the reported specificity ranged from 84–100% [6-16, 18-22]. The pooled sensitivity was 14% (95% CI, 9–20%; Q = 213.03, P < 0.01; I^2 = 93%), and the pooled specificity was 98% (95% CI, 96–99%; Q = 156.06, P < 0.01; I^2 = 90%). The LR+ and LR− were 6.3 (95% CI, 4.1–9.7) and 0.88 (95% CI, 0.84–0.93), respectively. The AUC of the SROC was 0.68 (95% CI, 0.64–0.72). The sensitivity forest plots, specificity forest plots, and SROC are shown in Figs 3A, 3B, and 3C, respectively.

Meta-analysis of the diagnostic accuracy of the anti-GP2 antibody (either IgG or IgA) for CD

A total of 1,818 CD patients and 2,188 controls were involved in this part of the meta-analysis. The diagnostic sensitivity of the anti-GP2 antibody (IgG or IgA) for CD ranged from 10–54%, and the reported

For peer review only - http://bmjopen.bmj.com/site/about/guidelines.xhtml

For peer review only

specificity ranged from 84–100% [6-12, 18, 19, 21]. The pooled sensitivity was 24% (95% CI, 18–32%; Q = 103.59, P < 0.01; I^2 = 90%), and the pooled specificity was 96% (95% CI, 93–97%; Q = 55.87, P < 0.01; I^2 = 82%). The LR+ and LR− were 5.4 (95% CI, 4.1–7.2) and 0.79 (95% CI, 0.74–0.85), respectively. The AUC of the SROC was 0.72 (95% CI, 0.68–0.76). The sensitivity forest plots, specificity forest plots, and SROC are shown in Figs 4A, 4B, and 4C, respectively.

Multiple regression analysis and exploration of threshold effect

Meta-regression analysis was conducted to explore possible sources of heterogeneity. The following co-variates were evaluated: the method of autoantibody detection, the manufacturer of detection kits, the region where the study was performed, and the QUADAS scores. Results indicated that the method of autoantibody detection led to heterogeneity. No other sources of heterogeneity were found.

In our study, none of the SROCs exhibited a “shoulder-arm” shape. In addition, none of the forest plots of pooled sensitivity or specificity showed a trend of negative correlation. Furthermore, the Spearman’s rank correlation (Spearman’s correlation coefficient and P-value for anti-GP2 antibody (IgG), anti-GP2 antibody (IgA), and anti-GP2 antibody (either IgG or IgA) were 0.525 and 0.025, 0.622 and 0.008, 0.761 and 0.007, respectively) did not show a strong positive correlation. These results indicated of no threshold effect.

Publication bias

The presence of a statistically significant slope coefficient (P < 0.05) is believed to indicate possible bias. We conducted funnel plots that represented a somewhat symmetric curve (Fig 5). The P-values of the slope coefficients for the anti-GP2 antibody using IgG, IgA, and either IgG or IgA were determined to be 0.11,

For peer review only - http://bmjopen.bmj.com/site/about/guidelines.xhtml

For peer review only

0.10, and 0.22, respectively, indicating that no publication bias was observed among the included studies.

Discussion

The diagnosis of CD currently relies on a triad of clinical characteristics: radiological features and endoscopic and histological assessment. However, common clinical manifestations are not specific to CD. Furthermore, endoscopic and histological assessments are invasive and may not be easily tolerated by patients and clinicians. Non-invasive assessments alone, such as radiological tests, may not be adequate for diagnosing CD. To date, anti-GP2 antibody is considering an emerging factor for diagnosis of CD. To our knowledge, this study is the first meta-analysis to provide precise and controlled data on the diagnostic performance of the anti-GP2 antibody in CD.

There were 17 eligible studies of high quality included in this meta-analysis (Supplementary Table 1). To explore the diagnostic performance of the anti-GP2 antibody IgG subtype, IgA subtype, and either the IgG or IgA subtype, we calculated statistics for each of these.

Generally, studies can be pooled together to calculate pooled statistics in diagnostic meta-analysis when there is no threshold effect. Results of our study suggest that there were no threshold effects in the recruited studies. Thus, we calculated pooled statistics. The AUC of the SROCs were also calculated for reference.

For the anti-GP2 antibody IgG subtype, the pooled specificity was high (97%), with a relatively low sensitivity (19%), indicating that the anti-GP2 antibody IgG subtype may identify controls better than patients with CD. The AUC (0.71) of the SROC implies that the diagnostic performance of the anti-GP2 antibody IgG subtype is not satisfactory enough for CD. Compared with the IgG subtype, the anti-GP2

For peer review only - http://bmjopen.bmj.com/site/about/guidelines.xhtml

antibody IgA subtype showed a higher specificity (98%), lower sensitivity (14%), and smaller AUC of the SROC (0.68). Considering both the IgG and IgA subtype in the CD diagnosis, the pooled sensitivity and the AUC of the SROC was higher (24% and 0.72), while the pooled specificity was lower (96%) than when only one subtype was used.

These results show that detecting both the IgG and IgA subtypes of the anti-GP2 antibody may result in better diagnostic performance for CD than detecting either the IgA or IgG subtype alone. However, high levels of heterogeneity were detected in both the pooled sensitivity and specificity of the anti-GP2 antibody when detecting both the IgG and IgA subtypes. Therefore, we conducted a meta-regression analysis for the anti-GP2 antibody. However, no significant heterogeneity was found among the manufacturer of detection kits, the region where the study was performed, or the QUADAS scores when detecting both the IgG and IgA subtypes. Interestingly, the method of autoantibody detection was confirmed to be the source of the heterogeneity in the diagnostic specificity of the anti-GP2 antibody (IgG) in CD.

Clinical phenotypes of CD patients are determined based on the Montreal Classification [23]. Specifically, CD is described by A, L, and B classifications. The A classification represents the age at diagnosis (A1, < 17 yr; A2, 17–40 yr; A3, > 40 yr), L represents the location of the disease (L1, ileal; L2, colonic; L3, ileocolonic; L4, upper gastrointestinal tract), and B represents the disease behavior (B1, non-stricturing, non-penetrating; B2, stricturing; B3, penetrating; P, perianal disease modifier). Recently, an association between the loss of tolerance to GP2 and the phenotype of CD disease in accordance with the Montreal classification has been reported. The anti-GP2 antibody was found to be more prevalent among CD patients with the A1, B2, L1, and L3 phenotypes when compared to those with the A2, A3, B1, B3, and

For peer review only - http://bmjopen.bmj.com/site/about/guidelines.xhtml

L2 phenotypes [6, 7, 12, 17, 18]. Differences among the phenotypes of CD patients recruited in the eligible studies might result in heterogeneity in our meta-analysis. However, most of the recruited studies did not provide detection ratios of anti-GP2 antibody among the different subtypes of CD, so we are unable to consider this in our meta-analysis.

Of note, the meta-analysis revealed positive likelihood ratios of above 5 for the anti-GP2 antibody. This means that the anti-GP2 antibody exhibits at least a 30% change in probability and, thus, at least a moderate effect on the posttest probability of CD. Thus, the anti-GP2 antibody can be used for the differentiation of CD patients from controls.

Some relevant unpublished studies with negative findings that meet our inclusion criteria might have been missed in our analysis. Therefore, funnel plots were used to detect possible publication bias. However, no publication bias was detected in the funnel plots. One limitation of this meta-analysis is that we did not compare the diagnostic accuracy of the anti-GP2 antibody with that of the anti-*Saccharomyces cerevisiae* antibody owing to a lack of relevant studies.

In conclusion, the anti-GP2 antibody is a specific marker of CD, and it can be used for the differentiation of CD patients from controls. However, the diagnostic performance of the anti-GP2 antibody cannot be definitively concluded owing to heterogeneity among studies that has not been fully explained. Further exploration of the prevalence of the anti-GP2 antibody among different clinical phenotypes of CD will provide a better understanding of its diagnostic performance.

Author contribution: LYZ designed the research, DCW, LWL and LJ collected and analysis the data, DCW wrote the paper, LYZ and ZSL help optimize the research and proofread the paper.

For peer review only - http://bmjopen.bmj.com/site/about/guidelines.xhtml

Funding statement:

This work was supported by funding from the National Natural Science Foundation of China Grants (81302591, 81601833, 81501841, 81172857, 81373188), the Research Special Fund for Public Welfare Industry of Health (201202004), the Chinese National High Technology Research and Development Program, Ministry of Science and Technology Grants (2011AA02A113), and the National Science Technology Pillar Program in the 12nd Five-year Plan (2014BAI07B00).

Competing interest: The authors declare that no competing interests exist.

Data sharing statement: All raw data are supplied in the Supplementary Table 1. All recorded information from the data extraction process, not included in the systematic review article, will be available on request.

Reference

[1] Lennard-Jones JE. Classification of inflammatory bowel disease. Scand J Gastroenterol Suppl. 1989;170:2-6.

[2] Laass MW, Roggenbuck D, Conrad K. Diagnosis and classification of Crohn's disease. Autoimmun Rev. 2014;13:467-71.

[3] Bossuyt X. Serologic markers in inflammatory bowel disease. Clin Chem. 2006;52:171-81.

[4] Roggenbuck D, Reinhold D, Wex T, Goihl A, von Arnim U, Malfertheiner P, et al. Autoantibodies to GP2, the major zymogen granule membrane glycoprotein, are new markers in Crohn's disease. Clin Chim Acta. 2011;412:718-724.

[5] Stöcker W, Otte M, Ulrich S, Normann D, Finkbeiner H, Stöcker K, et al. Autoimmunity to pancreatic juice in Crohn's disease. Results of an autoantibody screening in patients with chronic inflammatory bowel

For peer review only - http://bmjopen.bmj.com/site/about/guidelines.xhtml

disease. Scand J Gastroenterol Suppl. 1987;139:41-52.

[6] Pavlidis P, Komorowski L, Teegen B, Liaskos C, Koutsoumpas AL, Smyk DS, et al. Diagnostic and clinical significance of Crohn's disease-specific pancreatic anti-GP2 and anti-CUZD1 antibodies. Clin Chem Lab Med. 2016;54:249-256.

[7] Michaels MA, Jendrek ST, Korf T, Nitzsche T, Teegen B, Komorowski L, et al. Pancreatic Autoantibodies Against CUZD1 and GP2 Are Associated with Distinct Clinical Phenotypes of Crohn's Disease. Inflamm Bowel Dis. 2015;21:2864-2872.

[8] Roggenbuck D, Vermeire S, Hoffman I, Reinhold D, Schierack P, Goihl A, et al. Evidence of Crohn's disease-related anti-glycoprotein 2 antibodies in patients with celiac disease. Clin Chem Lab Med. 2015;53:1349-1357.

[9] Zhang S, Wu Z, Luo J, Ding X, Hu C, Li P, et al. Diagnostic Potential of Zymogen Granule Glycoprotein 2 Antibodies as Serologic Biomarkers in Chinese Patients With Crohn Disease. Medicine (Baltimore). 2015;94:e1654.

[10] Pavlidis P, Shums Z, Koutsoumpas AL, Milo J, Papp M, Umemura T, et al. Diagnostic and clinical significance of Crohn's disease-specific anti-MZGP2 pancreatic antibodies by a novel ELISA. Clin Chim Acta. 2015;441:176-181.

[11] Laass MW, Röber N, Range U, Noß L, Roggenbuck D, Conrad K. Loss and Gain of Tolerance to Pancreatic Glycoprotein 2 in Celiac Disease. PLoS One. 2015;10:e0128104.

[12] Papp M, Sipeki N, Tornai T, Altorjay I, Norman GL, Shums Z, et al. Rediscovery of the Anti-Pancreatic Antibodies and Evaluation of their Prognostic Value in a Prospective Clinical Cohort of

For peer review only - http://bmjopen.bmj.com/site/about/guidelines.xhtml

Crohn's Patients: The Importance of Specific Target Antigens [GP2 and CUZD1]. J Crohns Colitis. 2015;9:659-668.

[13] Kohoutova D, Drahosova M, Moravkova P, Rejchrt S, Bures J. Anti-Outer membrane protein C and anti-glycoprotein 2 antibodies in inflammatory bowel disease and their association with complicated forms of Crohn's disease. BMC Gastroenterol. 2014;14:190.

[14] Gross S, Bakker SF, van Bodegraven AA, van Hoogstraten IM, Gelderman KA, Bouma G, et al. Increased IgA glycoprotein-2 specific antibody titres in refractory celiac disease. J Gastrointestin Liver Dis. 2014;23:127-133.

[15] Komorowski L, Teegen B, Probst C, Aulinger-Stöcker K, Sina C, Fellermann K, et al. Autoantibodies against exocrine pancreas in Crohn's disease are directed against two antigens: the glycoproteins CUZD1 and GP2. J Crohns Colitis. 2013;7:780-790.

[16] Bonaci-Nikolic B, Spuran M, Andrejevic S, Nikolic M. Autoantibodies to GP2, the major zymogen granule membrane glycoprotein, in patients with gluten-sensitive enteropathy: a possible serological trap. Clin Chim Acta. 2012;413:822-823.

[17] Pavlidis P, Romanidou O, Roggenbuck D, Mytilinaiou MG, Al-Sulttan F, Liaskos C, et al. Ileal inflammation may trigger the development of GP2-specific pancreatic autoantibodies in patients with Crohn's disease. Clin Dev Immunol. 2012;2012:640835.

[18] Bogdanos DP, Roggenbuck D, Reinhold D, Wex T, Pavlidis P, von Arnim U, et al. Pancreatic-specific autoantibodies to glycoprotein 2 mirror disease location and behaviour in younger patients with Crohn's disease. BMC Gastroenterol. 2012;12:102.

For peer review only - http://bmjopen.bmj.com/site/about/guidelines.xhtml

[19] Op De Beéck K, Vermeire S, Rutgeerts P, Bossuyt X. Antibodies to GP2, the major zymogen granule membrane glycoprotein, in inflammatory bowel diseases. Gut. 2012;61:162-4; author reply 164-165.

[20] Pavlidis P, Forbes A, Bogdanos DP. Antibodies to glycoprotein 2 (GP2) in patients with inflammatory bowel diseases from UK. Clin Chim Acta. 2011;412:1163-1164.

[21] Roggenbuck D, Reinhold D, Wex T, Goihl A, von Arnim U, Malfertheiner P, et al. Autoantibodies to GP2, the major zymogen granule membrane glycoprotein, are new markers in Crohn's disease. Clin Chim Acta. 2011;412:718-724.

[22] Roggenbuck D, Hausdorf G, Martinez-Gamboa L, Reinhold D, Büttner T, Jungblut PR, et al. Identification of GP2, the major zymogen granule membrane glycoprotein, as the autoantigen of pancreatic antibodies in Crohn's disease. Gut. 2009;58:1620-1628.

[23] Satsangi J, Silverberg MS, Vermeire S, Colombel JF. The Montreal classification of inflammatory bowel disease: controversies, consensus, and implications. Gut. 2006;55:749-753.

Figure legend

Fig 1. Flow chart of studies included in the meta-analysis.

Fig 2. The forest plots and the summary receiver operating characteristic curves of anti-glycoprotein 2 antibody (IgG) for CD. A: Sensitivity forest plot; B: Specificity forest plot; C: Summary Receiver Operating Characteristic.

Fig 3. The forest plots and the summary receiver operating characteristic curves of anti-glycoprotein 2 antibody (IgA) for CD. A: Sensitivity forest plot; B: Specificity forest plot; C: Summary Receiver Operating Characteristic.

For peer review only

Fig 4. The forest plots and the summary receiver operating characteristic curves of anti-glycoprotein 2 antibody (either IgG or IgA) for CD. A: Sensitivity forest plot; B: Specificity forest plot; C: Summary Receiver Operating Characteristic.

Fig 5. Funnel plot of the publication bias for the anti-glycoprotein 2 antibody (IgG and IgA either). ESS is the abbreviation of effective sample size.

For peer review only

For peer review only - http://bmjopen.bmj.com/site/about/guidelines.xhtml

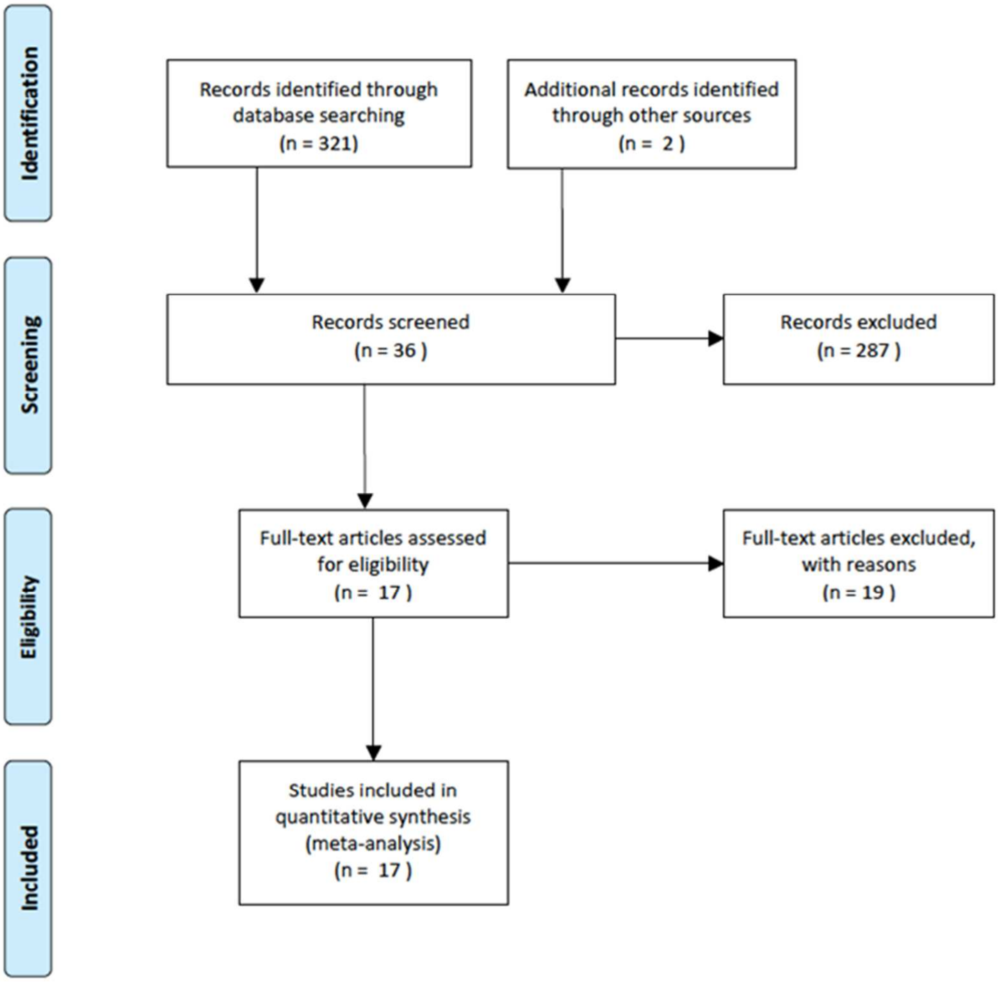

193x190mm (300 x 300 DPI)

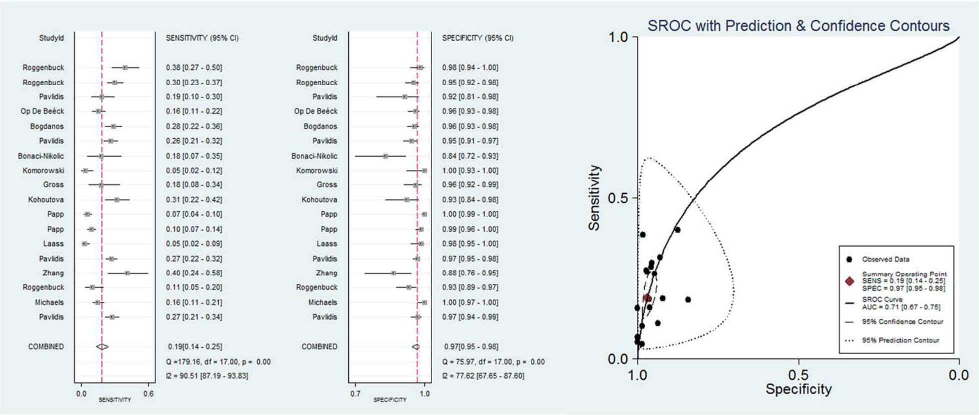

(A) (B) (C)

180x84mm (300 x 300 DPI)

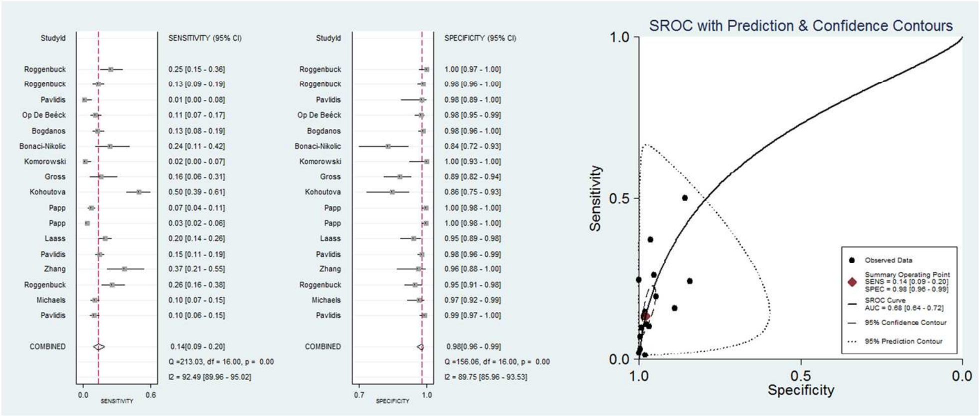

(A) (B) (C)

180x81mm (300 x 300 DPI)

For peer review only

For peer review only - http://bmjopen.bmj.com/site/about/guidelines.xhtml

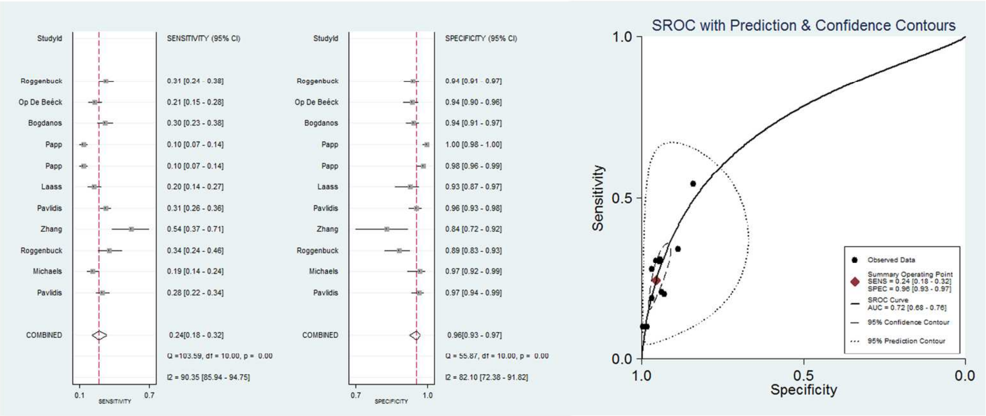

(A) (B) (C)

180x81mm (300 x 300 DPI)

For peer review only

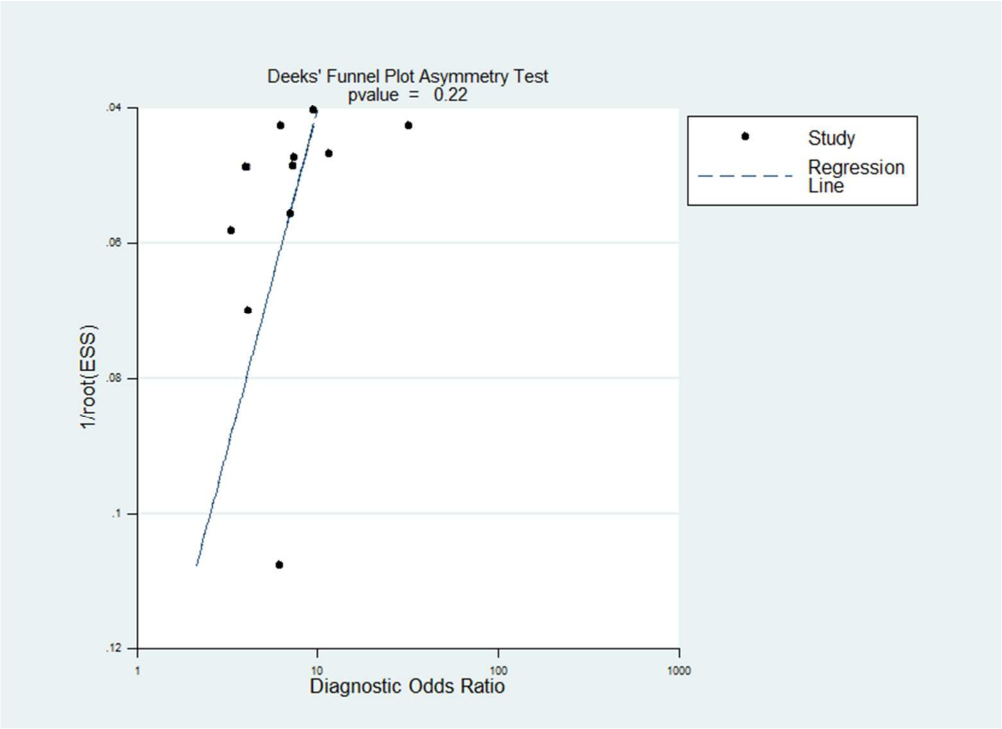

292x212mm (300 x 300 DPI)

For peer review only - http://bmjopen.bmj.com/site/about/guidelines.xhtml

The specific search algorithms of Pubmed

((anti-glycoprotein[All Fields] AND 2[All Fields] AND ("immunoglobulins"[MeSH Terms] OR "immunoglobulins"[All Fields] OR "antibody"[All Fields] OR "antibodies"[MeSH Terms] OR "antibodies"[All Fields])) OR (("glycoproteins"[MeSH Terms] OR "glycoproteins"[All Fields] OR "glycoprotein"[All Fields]) AND 2[All Fields] AND ("autoantibodies"[MeSH Terms] OR "autoantibodies"[All Fields])) OR (("autoantibodies"[MeSH Terms] OR "autoantibodies"[All Fields]) AND ("glycoproteins"[MeSH Terms] OR "glycoproteins"[All Fields] OR "glycoprotein"[All Fields]) AND 2[All Fields])) AND ("crohn disease"[MeSH Terms] OR ("crohn"[All Fields] AND "disease"[All Fields]) OR "crohn disease"[All Fields] OR ("crohn's"[All Fields] AND "disease"[All Fields]) OR "crohn's disease"[All Fields])

For peer review only

Supplementary Table 1 Characteristics of studies included in the meta-analysis of diagnostic performance of anti-glycoprotein 2 antibody in CD

Reference	Year	Region	Author	Manufacturer	Method	Ig subtype	Case	Control	TP	FP	FN	TN	QUADAS score
						IgG			58	7	154	242	
[6]	2016	United Kingdom	Pavlidis et al.	In-house kit	IIF	IgA	212	249	21	2	191	247	12
						either			59	8	153	241	
						IgG			35	0	189	126	
[7]	2015	Germany	Michaels et al.	Euroimmune	IIF	IgA	224	126	23	4	201	122	12
						either			42	4	182	122	
						IgG			8	11	65	158	
[8]	2015	Germany	Roggenbuck et al.	GA Generic Assays	ELISA	IgA	73	169	19	8	54	161	11
						either			25	19	48	150	
						IgG			14	7	21	49	
[9]	2015	China	Zhang et al.	GA Generic Assays	ELISA	IgA	35	56	13	2	22	54	11
						either			19	9	16	47	
						IgG	323	509	87	15	236	494	
[10]	2015	United Kingdom	Pavlidis et al.	Inova Diagnostics	ELISA	IgA			48	10	275	499	12
						either	323	294	99	13	224	281	
						IgG			8	2	166	127	
[11]	2015	Germany	Laass et al.	GA Generic Assays	ELISA	IgA	174	129	34	7	140	122	10
						either			35	9	139	120	

For peer review only

Supplementary Table 1 Characteristics of studies included in the meta-analysis of diagnostic performance of anti-glycoprotein 2 antibody in CD (continued)

Reference	Year	Region	Author	Manufacturer	Method	Ig subtype	Case	Control	TP	FP	FN	TN	QUADAS score
[12]	2015	Hungary	Papp et al.	GA Generic Assays	ELISA	IgG	266	283	27	4	239	279	12
						IgA			9	1	257	282	
						either			27	5	239	278	
				Euroimmune	IIF	IgG			18	0	248	283	
						IgA			19	1	247	282	
						either			27	1	239	282	
[13]	2014	Czech Republic	Kohoutova et al.	GA Generic Assays	ELISA	IgG	86	70	27	5	59	65	6
						IgA			43	10	43	60	
						either NA			/	/	/	/	
[14]	2014	Netherlands	Gross et al.	GA Generic Assays	ELISA	IgG	38	136	7	5	31	131	11
						IgA			6	15	32	121	
						either NA			/	/	/	/	
[15]	2013	Germany	Komorowski et al.	In-house kit	IIF	IgG	96	50	5	0	91	50	10
						IgA			2	0	94	50	
						either NA			/	/	/	/	
[16]	2012	Serbia	Bonaci-Nikolic et al.	GA Generic Assays	ELISA	IgG	33	57	6	9	27	48	11
						IgA			8	9	25	48	
						either NA			/	/	/	/	

For peer review only

Supplementary Table 1 Characteristics of studies included in the meta-analysis of diagnostic performance of anti-glycoprotein 2 antibody in CD (continued)

Reference	Year	Region	Author	Manufacturer	Method	Ig subtype	Case	Control	TP	FP	FN	TN	QUADAS score
						IgG			59	16	166	284	
[17]	2012	United Kingdom	Pavlidis et al.	GA Generic Assays	ELISA	IgA NA	225	300	/	/	/	/	11
						either NA			/	/	/	/	
						IgG			48	14	121	313	
[18]	2012	Germany	Bogdanos et al.	GA Generic Assays	ELISA	IgA	169	327	22	5	147	322	11
						either			51	18	118	309	
						IgG			26	11	138	282	
[19]	2012	Belgium	Op De Beéck et al.	GA Generic Assays	ELISA	IgA	164	293	18	7	146	286	12
						either			34	18	130	275	
						IgG			13	4	57	46	
[20]	2011	United Kingdom	Pavlidis et al.	GA Generic Assays	ELISA	IgA	70	50	1	1	69	49	6
						either NA			/	/	/	/	
						IgG			53	12	125	250	
[21]	2011	Germany	Roggenbuck et al.	In-house kit	ELISA	IgA	178	262	24	4	154	258	11
						either			55	15	123	247	
						IgG			28	2	45	116	
[22]	2009	Germany	Roggenbuck et al.	In-house kit	IIF	IgA	73	118	18	0	55	118	11
						either NA			/	/	/	/	

Note: IIF: indirect immunofluorescence; ELISA: enzyme-linked immuno sorbent assay; NA: not applicable; TP: true positive; FP: false positive; TN: true negative; FN: false negative.

For peer review only

PRISMA 2009 Checklist

Section/Topic	#	Checklist Item	Reported on Page #
**TITLE**			
Title	1	Identify the report as a systematic review, meta-analysis, or both.	1
**ABSTRACT**			
Structured summary	2	Provide a structured summary including, as applicable: background; objectives; data sources; study eligibility criteria, participants, and interventions; study appraisal and synthesis methods; results; limitations; conclusions and implications of key findings; systematic review registration number.	2
**INTRODUCTION**			
Rationale	3	Describe the rationale for the review in the context of what is already known.	4-5
Objectives	4	Provide an explicit statement of questions being addressed with reference to participants, interventions, comparisons, outcomes, and study design (PICOS).	5
**METHODS**			
Protocol and registration	5	Indicate if a review protocol exists, if and where it can be accessed (e.g., Web address), and, if available, provide registration information including registration number.	Not applicable
Eligibility criteria	6	Specify study characteristics (e.g., PICOS, length of follow-up) and report characteristics (e.g., years considered, language, publication status) used as criteria for eligibility, giving rationale.	5-6
Information sources	7	Describe all information sources (e.g., databases with dates of coverage, contact with study authors to identify additional studies) in the search and date last searched.	5
Search	8	Present full electronic search strategy for at least one database, including any limits used, such that it could be repeated.	5
Study selection	9	State the process for selecting studies (i.e., screening, eligibility, included in systematic review, and, if applicable, included in the meta-analysis).	5-6
Data collection process	10	Describe method of data extraction from reports (e.g., piloted forms, independently, in duplicate) and any processes for obtaining and confirming data from investigators.	6
Data items	11	List and define all variables for which data were sought (e.g., PICOS, funding sources) and any assumptions and simplifications made.	6
Risk of bias in individual studies	12	Describe methods used for assessing risk of bias of individual studies (including specification of whether this was done at the study or outcome level), and how this information is to be used in any data synthesis.	Not applicable
Summary measures	13	State the principal summary measures (e.g., risk ratio, difference in means).	6
Synthesis of results	14	Describe the methods of handling data and combining results of studies, if done, including measures of consistency (e.g., I^2 for each meta-analysis.	6-7

For peer review only

PRISMA 2009 Checklist

Section/Topic	#	Checklist Item	Reported on Page #
Risk of bias across studies	15	Specify any assessment of risk of bias that may affect the cumulative evidence (e.g., publication bias, selective reporting within studies).	7
Additional analyses	16	Describe methods of additional analyses (e.g., sensitivity or subgroup analyses, meta-regression), if done, indicating which were pre-specified.	7
**RESULTS**			
Study selection	17	Give numbers of studies screened, assessed for eligibility, and included in the review, with reasons for exclusions at each stage, ideally with a flow diagram.	7
Study characteristics	18	For each study, present characteristics for which data were extracted (e.g., study size, PICOS, follow-up period) and provide the citations.	7
Risk of bias within studies	19	Present data on risk of bias of each study and, if available, any outcome level assessment (see item 12).	Not applicable
Results of individual studies	20	For all outcomes considered (benefits or harms), present, for each study: (a) simple summary data for each intervention group (b) effect estimates and confidence intervals, ideally with a forest plot.	8-9
Synthesis of results	21	Present the main results of the review. If meta-analyses done, include for each, confidence intervals and measures of consistency.	8-9
Risk of bias across studies	22	Present results of any assessment of risk of bias across studies (see Item 15).	Not applicable
Additional analysis	23	Give results of additional analyses, if done (e.g., sensitivity or subgroup analyses, meta-regression [see Item 16]).	9-10
**DISCUSSION**			
Summary of evidence	24	Summarize the main findings including the strength of evidence for each main outcome; consider their relevance to key groups (e.g., healthcare providers, users, and policy makers).	10-11
Limitations	25	Discuss limitations at study and outcome level (e.g., risk of bias), and at review-level (e.g., incomplete retrieval of identified research, reporting bias).	12
Conclusions	26	Provide a general interpretation of the results in the context of other evidence, and implications for future research.	12
**FUNDING**			
Funding	27	Describe sources of funding for the systematic review and other support (e.g., supply of data); role of funders for the systematic review.	13

From: Moher D, Liberati A, Tetzlaff J, Altman DG, The PRISMA Group (2009). Preferred Reporting Items for Systematic Reviews and Meta-Analyses: The PRISMA Statement. PLoS Med 6(6): e1000097. doi:10.1371/journal.pmed1000097

For peer review only - http://bmjopen.bmj.com/site/about/guidelines.xhtml

PRISMA 2009 Checklist

For more information, visit: www.prisma-statement.org.

For peer review only

For peer review only - http://bmjopen.bmj.com/site/about/guidelines.xhtml

BMJ Open

Diagnostic value of the anti-glycoprotein-2 antibody for Crohn's disease: A PRISMA-compliant systematic review and meta-analysis

Journal:	*BMJ Open*
Manuscript ID	bmjopen-2016-014843.R2
Article Type:	Research
Date Submitted by the Author:	17-Apr-2017
Complete List of Authors:	Deng, Chuiwen; Peking Union Medical College Hospital, Chinese Academy of Medical Sciences and Peking Union Medical College, Rheumatology and Clinical Immunology<br>Li, Wenli<br>Li, Jing<br>Zhang, Shulan<br>Li, Yongzhe; Peking Union Medical College Hospital,Chinese Academy of Medical Sciences and Peking Union Medical College, Rheumatology and Clinical Immunology
**Primary Subject Heading**:	Gastroenterology and hepatology
Secondary Subject Heading:	Gastroenterology and hepatology
Keywords:	Inflammatory bowel disease < GASTROENTEROLOGY, Gastroenterology < INTERNAL MEDICINE, IMMUNOLOGY

SCHOLARONE™
Manuscripts

For peer review only

Diagnostic value of the anti-glycoprotein-2 antibody for Crohn's disease: A PRISMA-compliant systematic review and meta-analysis

Chuiwen Deng,[1¶] Wenli Li,[2¶] Jing Li,[3¶] Shulan Zhang,[1] Yongzhe Li,[1*]

[1]Department of Rheumatology and Clinical Immunology, Peking Union Medical College Hospital, Chinese Academy of Medical Sciences & Peking Union Medical College, Key Laboratory of Rheumatology and Clinical Immunology, Ministry of Education, Beijing, China.

[2]Department of Rheumatology, China-Japan Friendship Hospital, Beijing, China.

[3]Clinical laboratory, Peking university international hospital, Beijing, China.

[*]Correspondence author

Email address: yongzhelipumch@126.com (LYZ)

[¶]These authors are the co-first authors on this study.

Short title: Anti-GP2 antibody in CD, a meta-analysis

For peer review only - http://bmjopen.bmj.com/site/about/guidelines.xhtml

Abstract

Objectives: To perform a meta-analysis to evaluate the diagnostic performance of the anti-glycoprotein-2 (GP2) antibody for Crohn's disease (CD).

Methods: Three databases (EMBASE, ISI Web of Knowledge, and PubMed) were systematically searched. There were 17 eligible studies included in the meta-analysis. A total of 2,439 CD patients and 3,184 controls were involved in these studies. STATA 11.2 and Meta-DiSc 1.4 were used to perform the meta-analysis.

Results: The area under the summary receiver operating characteristic curve was 0.68–0.72. The pooled diagnostic sensitivity of the anti-GP2 antibody ranged from 14 to 24%, and the specificity was 96–98%.

Conclusions: The anti-GP2 antibody is a specific biomarker for CD, and further exploration of its prevalence among different clinical phenotypes of CD will provide a better understanding of its diagnostic performance.

Article summary

Strengths and limitations of this study:

1. This meta-analysis was prepared according to the Preferred Reporting Items for Systematic Review and Meta-Analysis.

2. The methods that were applied in this meta-analysis included evaluations of the threshold effect, pooled statistics, operating characteristic curve, publication bias, and other sources of heterogeneity.

3. The full-text screening, data extraction, and analysis of the included studies were conducted by two reviewers independently.

For peer review only

For peer review only - http://bmjopen.bmj.com/site/about/guidelines.xhtml

4. This is the first systematic review and meta-analysis to evaluate the diagnostic performance of the anti-glycoprotein-2 antibody for Crohn's disease.

5. Some of the included studies were of different aims from the meta-analysis for few researches were undertaken to precisely evaluate the diagnostic performance of anti-GP2 antibody, and this strategy may introduce potential heterogeneity.

Keywords: Anti-glycoprotein 2 antibody; Crohn's disease; Diagnostic performance; Meta-analysis

For peer review only

For peer review only - http://bmjopen.bmj.com/site/about/guidelines.xhtml

Introduction

Crohn's disease (CD) is a chronic inflammatory disorder of the gastrointestinal tract. Usually, the diagnosis of CD is based on current standard clinical, radiological, endoscopic, and histological criteria [1]. However, clinical symptoms and radiological evidence are not specific, while endoscopic and histological tests are invasive procedures that are not always accepted by patients. A precise method for diagnosing CD and differentiating it from ulcerative colitis (UC) is urgently needed.

There is cumulative evidence from cross-sectional and longitudinal studies to support the value of serological markers in the diagnosis, differential diagnosis, and prediction of disease course in CD [2]. The most widely used serological marker for CD is the anti-*Saccharomyces cerevisiae* antibody. However, its diagnostic performance in CD has not achieved sufficiently high sensitivity or specificity for clinical needs [3]. Recently, pancreatic autoantibody (PAB) has emerged as a potential diagnostic marker for CD [4]. The presence of PAB was first identified by indirect immunofluorescence [5]. Recently, the target antigens of PAB have been identified as the pancreatic major granule glycoprotein 2 (GP2) of the zymogen granule membrane and the CUZD1 protein [4]. The anti-GP2 antibody can be detected using an enzyme-linked immunosorbent assay in routine practice.

Over the past decade, numerous studies have evaluated the anti-GP2 antibody for its ability to accurately diagnose CD. However, inconsistent conclusions relating to the diagnostic performance of anti-GP2 antibody have been drawn [4, 6-21]. The reported diagnostic sensitivity of anti-GP2 antibody (IgG) ranges from 5 to 40%, and the reported diagnostic specificity ranges from 84 to 100%. The IgA

For peer review only - http://bmjopen.bmj.com/site/about/guidelines.xhtml

subtype of the anti-GP2 antibody is also considered to be potentially valuable in CD diagnosis, but the reported sensitivities (1–50%) and specificities (84–100%) also show high variation. In addition, some researchers have proposed that combing the results of both IgG and IgA subtype of the anti-GP2 antibody would improve its diagnostic value of CD, while others have disagreed. Lastly, methodological discrepancies, including the subtypes of CD tested, the method of autoantibody detection, and the manufacturer of the detection kits might influence the diagnostic value of the anti-GP2 antibody, and these should be investigated.

In order to verify the diagnostic performance of the anti-GP2 antibody in patients with CD and to determine the factors that influence the results of anti-GP2 antibody testing, we performed the present systematic review and meta-analysis

Methods

Literature search

Studies were identified in EMBASE, ISI Web of Knowledge, and PubMed databases. To retrieve all relevant publications related to anti-GP2 antibody and CD, we searched for the follow terms: “anti-glycoprotein 2 antibody”, “glycoprotein 2 autoantibodies”, and “autoantibodies to glycoprotein 2”, combined with “Crohn’s disease” and “CD”. No limits were placed on ethnicity or geographic region, and all documents were included up to June 2016. The specific PubMed search algorithm is provided in Supplementary File 1. Additional relevant references cited in searched articles were also selected, if any. All analyses in this systemic review were based on previously published studies, and thus no ethical approval or patient consent was required.

For peer review only - http://bmjopen.bmj.com/site/about/guidelines.xhtml

Eligibility criteria

The following criteria were used to determine eligibility for inclusion: (1) studies that assessed the diagnostic accuracy of the anti-GP2 antibody for CD were included; (2) studies with sufficient data to construct two-by-two tables were included; (3) the control groups set for the CD patients should fulfill one of the following: people with complaints (abdominal pain, diarrhea, and ileus, etc.) that made CD a relevant diagnostic possibility; patients diagnosed with the diseases that need to be differential with CD, such as ulcerative colitis, irritable bowl syndrome, ischemic bowel disease and so on. (4) all published languages were included; and (5) studies based on animals or cell cultures or case reports without subsequent publication in full text were excluded. In the case of overlapping studies, only the study with the largest sample size was included in our analysis.

Data extraction

Data were extracted from all selected studies by two independent investigators. Inter-researcher disagreements were resolved by consensus or by a third investigator. The following data were collected from each selected study: first author's name; publication year; country in which the study was performed; and study results. Formally, the combined test of IgG and IgA subtypes of the anti-GP2 antibody is taken as positive if one or both tests is positive (logical OR of positive results); it is negative if both tests are negative (logical AND of negativity). Basing on this knowledge, the 'either IgG or IgA' phrase of this meta-analysis means that either IgG or IgA subtypes of the anti-GP2 antibody is found to be present (abnormal). Study quality was assessed using the Quality Assessment of Diagnostic Accuracy Studies (QUADAS) tool. Authors of the identified studies were contacted via e-mail if further study details were

For peer review only

For peer review only - http://bmjopen.bmj.com/site/about/guidelines.xhtml

needed.

Statistical analysis

Statistical analysis was performed using STATA 11.2 (Stata Corporation, College Station, TX, USA) and Meta-DiSc 1.4 (Unit of Clinical Biostatistics, Ramon y Cajal Hospital, Madrid, Spain). Potential important differences in the results of the individual studies are frequently referred to as heterogeneity. In this meta-analysis, the heterogeneity among studies was evaluated by Cochrane's *Q*-statistic as well as by the I^2-statistic. A *Q*-statistic P-value > 0.10 indicated lack of heterogeneity among studies. $I^2 < 25\%$ was considered low heterogeneity, 25–50% moderate, and > 50% indicated a high degree of heterogeneity. Finally, the overall or pooled sensitivity, specificity, positive likelihood ratio (LR+), negative likelihood ratio (LR–), and their 95% confidence intervals (Cis) were obtained by a random-effects or a fixed-effects model in the presence ($P \leq 0.10$ or $I^2 > 50\%$) or absence ($P > 0.10$ and $I^2 \leq 50\%$) of heterogeneity, respectively. The area under the summary receiver operating characteristic (SROC) curve represented the overall performance of the detection method. Besides the area under the curve (AUC), the ROC analysis also provide estimates of uncertainty that includes a 95% confidence region which indicates the precision of studies in the pooled estimate, and a 95% prediction region around the summary points which illustrate the amount of between study variation. A P-value < 0.05 (two-sided) was considered significant. Threshold effects occur when different cut-off values are applied in different studies, leading to heterogeneity of the results. A Spearman's rank correlation calculating between the logarithm of sensitivity and the logarithm of (1 – specificity) across source studies was performed to test for threshold effects. Evaluation of publication bias was also undertaken.

For peer review only - http://bmjopen.bmj.com/site/about/guidelines.xhtml

Results

Literature search

Electronic and manual searches yielded a total of 323 potentially eligible articles. A flow chart of the screening of articles for meta-analysis is illustrated in Fig 1. There were 287 articles that were excluded based on screening of the titles and abstracts. A further 19 full-text articles were excluded as they were not related to our subject. Finally, a total of 17 eligible studies were included in the meta-analysis [4, 6-21].

Study characteristics

The characteristics of the 17 studies are summarized in Supplementary Table 1. A total of 2,439 CD patients and 3,184 controls were involved in these studies. With regard to the geographic location of the studies, seven were carried out in Germany [4, 7, 8, 11, 15, 18, 21], four in the United Kingdom [6, 10, 17, 20], and one each in Serbia, the Netherlands, Hungary, the Czech Republic, Belgium, and China [9, 12, 13, 14, 16, 19]. Assessment using QUADAS indicated that the studies were of median to high quality, with positive results for at least 6/14 items.

Meta-analysis of the diagnostic accuracy of the anti-GP2 antibody (IgG) for CD

A total of 2,439 CD patients and 3,191 controls were involved in this part of the meta-analysis. The diagnostic sensitivity of the anti-GP2 antibody (IgG) for CD ranged from 5–38%, and the reported specificity ranged from 84–100% [4, 6-21]. The diagnostic odds ratio was 7 (95% CI, 5–11; Q = 87.68, P < 0.01; I^2 = 98%). The pooled sensitivity was 19% (95% CI, 14–25%; Q = 179.16, P < 0.01; I^2 = 91%), and the pooled specificity was 97% (95% CI, 94–98%; Q = 75.97, P < 0.01; I^2 = 78%). The LR+ and LR− were 6.1 (95% CI, 4.1–9.1) and 0.84 (95% CI, 0.79–0.89), respectively. The AUC of the SROC was 0.71 (95%

For peer review only

CI, 0.67–0.75). The 95% confidence region of the SROC was narrow and small, increasing the precision of studies in the pooled estimate. The 95% prediction region of the SROC was broad and large, suggesting heterogeneity between studies. The sensitivity forest plots, specificity forest plots, and SROC are shown in Figs 2A, 2B, and 2C, respectively.

Meta-analysis of the diagnostic accuracy of the anti-GP2 antibody (IgA) for CD

A total of 2,214 CD patients and 2,894 controls were involved in this part of the meta-analysis. The diagnostic sensitivity of the anti-GP2 antibody (IgA) for CD ranged from 1–50%, and the reported specificity ranged from 84–100% [4, 6-16, 18-21]. The pooled sensitivity was 14% (95% CI, 9–20%; Q = 213.03, P < 0.01; I^2 = 93%), and the pooled specificity was 98% (95% CI, 96–99%; Q = 156.06, P < 0.01; I^2 = 90%). The LR+ and LR− were 6.3 (95% CI, 4.1–9.7) and 0.88 (95% CI, 0.84–0.93), respectively. The AUC of the SROC was 0.68 (95% CI, 0.64–0.72). The 95% confidence region of the SROC was narrow and small, increasing the precision of studies in the pooled estimate. The 95% prediction region of the SROC was broad and large, suggesting heterogeneity between studies. The sensitivity forest plots, specificity forest plots, and SROC are shown in Figs 3A, 3B, and 3C, respectively.

Meta-analysis of the diagnostic accuracy of the anti-GP2 antibody (either IgG or IgA) for CD

A total of 1,818 CD patients and 2,195 controls were involved in this part of the meta-analysis. The diagnostic sensitivity of the anti-GP2 antibody (IgG or IgA) for CD ranged from 10–54%, and the reported specificity ranged from 84–100% [4, 6-12, 18, 19]. The pooled sensitivity was 24% (95% CI, 18–32%; Q = 103.59, P < 0.01; I^2 = 90%), and the pooled specificity was 96% (95% CI, 93–97%; Q = 55.87, P < 0.01; I^2 = 82%). The LR+ and LR− were 5.4 (95% CI, 4.1–7.2) and 0.79 (95% CI, 0.74–0.85), respectively. The

For peer review only - http://bmjopen.bmj.com/site/about/guidelines.xhtml

AUC of the SROC was 0.72 (95% CI, 0.68–0.76). The 95% confidence region of the SROC was narrow and small, increasing the precision of studies in the pooled estimate. The 95% prediction region of the SROC was broad and large, suggesting heterogeneity between studies. The sensitivity forest plots, specificity forest plots, and SROC are shown in Figs 4A, 4B, and 4C, respectively.

Multiple regression analysis and exploration of threshold effect

Meta-regression analysis was conducted to explore possible sources of heterogeneity. The following co-variates were evaluated: the method of autoantibody detection (indirect immunofluorescence and enzyme-linked immuno sorbent assay) and the manufacturer of detection kits (Euroimmune, GA Generic, Inova Diagnostics or in-house kits), the region where the study was performed, and the QUADAS scores. Results indicated that the method of autoantibody detection led to heterogeneity. No other sources of heterogeneity were found.

The Spearman's correlation coefficient for anti-GP2 antibody (IgG), anti-GP2 antibody (IgA), and anti-GP2 antibody (either IgG or IgA) were 0.525 ($P = 0.025$), 0.622 ($P = 0.008$), 0.761 ($P = 0.007$), respectively. Basing on these characteristics, a fairly strong and significant correlation is found in each of the three cases, indicating existence of threshold effect.

Publication bias

The presence of a statistically significant slope coefficient ($P < 0.05$) is believed to indicate possible bias. We conducted funnel plots that represented a somewhat symmetric curve (Fig 5). The P-values of the slope coefficients for the anti-GP2 antibody using IgG, IgA, and either IgG or IgA were determined to be 0.11, 0.10, and 0.22, respectively, indicating that no publication bias was observed among the included studies.

For peer review only - http://bmjopen.bmj.com/site/about/guidelines.xhtml

Discussion

The diagnosis of CD currently relies on a triad of clinical characteristics: radiological features and endoscopic and histological assessment. However, common clinical manifestations are not specific to CD. Furthermore, endoscopic and histological assessments are invasive and may not be easily tolerated by patients and clinicians. Non-invasive assessments alone, such as radiological tests, may not be adequate for diagnosing CD. To date, anti-GP2 antibody is considering an emerging factor for diagnosis of CD. To our knowledge, this study is the first meta-analysis to provide precise and controlled data on the diagnostic performance of the anti-GP2 antibody in CD.

There were 17 eligible studies of high quality included in this meta-analysis (Supplementary Table 1). To explore the diagnostic performance of the anti-GP2 antibody IgG subtype, IgA subtype, and either the IgG or IgA subtype, we calculated statistics for each of these.

Generally, studies can be pooled together to calculate pooled statistics in diagnostic meta-analysis when there is no threshold effect. If not, the AUC of the SROCs should be calculated instead. Results of our study suggest that there were fairly strong and significant threshold effects in the recruited studies. Thus, we calculated the AUC of the SROCs. The pooled statistics were also calculated for reference.

For the anti-GP2 antibody IgG subtype, the AUC (0.71) of the SROC implies that the diagnostic performance of the anti-GP2 antibody IgG subtype is not satisfactory enough for CD. The pooled specificity of the anti-GP2 antibody IgG subtype was high (97%), with a relatively low sensitivity (19%). Compared with the IgG subtype, the anti-GP2 antibody IgA subtype showed a smaller AUC of the SROC (0.68), higher specificity (98%), and lower sensitivity (14%). Considering both the IgG and IgA subtype in

For peer review only - http://bmjopen.bmj.com/site/about/guidelines.xhtml

For peer review only

the CD diagnosis, the pooled sensitivity and the AUC of the SROC was higher (24% and 0.72), while the pooled specificity was lower (96%) than when only one subtype was used.

As we previously said that CD symptoms and manifestations are all non-specific. In other words, a specific test is needed for CD diagnosis. The results mentioned-above showed that anti-GP2 antibody was very specific for CD. Especially in the high specificity-low sensitivity situation we are dealing with, CD is more reliably identified than controls by anti-GP2 antibody. Admittedly, this occurs at the price of allowing identification of only a small fraction of CD cases because so the majority are false negatives.

In addition, these results show that testing for both the IgG and IgA subtypes of the anti-GP2 antibody may result in better diagnostic performance for CD than either the IgA or IgG subtype alone. However, high levels of heterogeneity were detected in among the prediction regions of the SROC, pooled sensitivity and specificity of the anti-GP2 antibody when testing for both the IgG and IgA subtypes. Therefore, we conducted a meta-regression analysis for the anti-GP2 antibody. However, no significant heterogeneity was found among the manufacturer of detection kits, the region where the study was performed, or the QUADAS scores when testing for both the IgG and IgA subtypes. Interestingly, the method of autoantibody detection was confirmed to be the source of the heterogeneity in the diagnostic specificity of the anti-GP2 antibody (IgG) in CD.

Clinical phenotypes of CD patients are determined based on the Montreal Classification [22]. Specifically, CD is described by A, L, and B classifications. The A classification represents the age at diagnosis (A1, < 17 yr; A2, 17–40 yr; A3, > 40 yr), L represents the location of the disease (L1, ileal; L2, colonic; L3, ileocolonic; L4, upper gastrointestinal tract), and B represents the disease behavior (B1, non-

For peer review only - http://bmjopen.bmj.com/site/about/guidelines.xhtml

stricturing, non-penetrating; B2, stricturing; B3, penetrating; P, perianal disease modifier). Recently, an association between the loss of tolerance to GP2 and the phenotype of CD disease in accordance with the Montreal classification has been reported. The anti-GP2 antibody was found to be more prevalent among CD patients with the A1, B2, L1, and L3 phenotypes when compared to those with the A2, A3, B1, B3, and L2 phenotypes [6, 7, 12, 17, 18]. Differences among the phenotypes of CD patients recruited in the eligible studies might result in heterogeneity in our meta-analysis. However, most of the recruited studies did not provide detection ratios of anti-GP2 antibody among the different subtypes of CD, so we are unable to consider this in our meta-analysis.

Of note, the meta-analysis revealed positive likelihood ratios of above 5 for the anti-GP2 antibody. This means that the anti-GP2 antibody exhibits at least a 30% change in probability and, thus, at least a moderate effect on the posttest probability of CD. Thus, the anti-GP2 antibody can be used for the differentiation of CD patients from controls.

Some relevant unpublished studies with negative findings that meet our inclusion criteria might have been missed in our analysis. Therefore, funnel plots were used to detect possible publication bias. And no publication bias was detected in the funnel plots.

There are two limitations of this meta-analysis. The PRISMA checklist assumes that the source studies should have the same aim as the meta-analysis. In this research, some of the included studies were investigations with different aims. Three of them concern subtypes of CD [7, 13, 18]; two have attention on refractory cases [14] or development of tolerance [11]; three focus on molecular processes [15, 17, 21]. Some studies may be published for disappointing results or other reasons [10, 16]. Up to date, few

For peer review only

researches were undertaken precisely to confirm the diagnostic value of anti-GP2 antibody, so we selected the current studies that help us evaluate this clinical index with potential value earlier. And this strategy may introduce potential heterogeneity. The other limitation of this meta-analysis is that we did not compare the diagnostic accuracy of the anti-GP2 antibody with that of the anti-*Saccharomyces cerevisiae* antibody owing to a lack of relevant studies.

In conclusion, the anti-GP2 antibody is a specific marker of CD, and it can be used for the differentiation of CD patients from controls. However, the diagnostic performance of the anti-GP2 antibody cannot be definitively concluded owing to heterogeneity among studies that has not been fully explained. Further exploration of the prevalence of the anti-GP2 antibody among different clinical phenotypes of CD will provide a better understanding of its diagnostic performance.

Author contribution: LYZ designed the research, DCW, LWL and LJ collected and analysis the data, DCW wrote the paper, LYZ and ZSL help optimize the research and proofread the paper.

Funding statement:

This work was supported by funding from the National Natural Science Foundation of China Grants (81302591, 81601833, 81501841, 81172857, 81373188), the Research Special Fund for Public Welfare Industry of Health (201202004), the Chinese National High Technology Research and Development Program, Ministry of Science and Technology Grants (2011AA02A113), and the National Science Technology Pillar Program in the 12nd Five-year Plan (2014BAI07B00).

Competing interest: The authors declare that no competing interests exist.

Data sharing statement: All raw data are supplied in the Supplementary Table 1. All recorded information

For peer review only - http://bmjopen.bmj.com/site/about/guidelines.xhtml

from the data extraction process, not included in the systematic review article, will be available on request.

Reference

[1] Lennard-Jones JE. Classification of inflammatory bowel disease. Scand J Gastroenterol Suppl. 1989;170:2-6.

[2] Laass MW, Roggenbuck D, Conrad K. Diagnosis and classification of Crohn's disease. Autoimmun Rev. 2014;13:467-71.

[3] Bossuyt X. Serologic markers in inflammatory bowel disease. Clin Chem. 2006;52:171-81.

[4] Roggenbuck D, Reinhold D, Wex T, Goihl A, von Arnim U, Malfertheiner P, et al. Autoantibodies to GP2, the major zymogen granule membrane glycoprotein, are new markers in Crohn's disease. Clin Chim Acta. 2011;412:718-724.

[5] Stöcker W, Otte M, Ulrich S, Normann D, Finkbeiner H, Stöcker K, et al. Autoimmunity to pancreatic juice in Crohn's disease. Results of an autoantibody screening in patients with chronic inflammatory bowel disease. Scand J Gastroenterol Suppl. 1987;139:41-52.

[6] Pavlidis P, Komorowski L, Teegen B, Liaskos C, Koutsoumpas AL, Smyk DS, et al. Diagnostic and clinical significance of Crohn's disease-specific pancreatic anti-GP2 and anti-CUZD1 antibodies. Clin Chem Lab Med. 2016;54:249-256.

[7] Michaels MA, Jendrek ST, Korf T, Nitzsche T, Teegen B, Komorowski L, et al. Pancreatic Autoantibodies Against CUZD1 and GP2 Are Associated with Distinct Clinical Phenotypes of Crohn's Disease. Inflamm Bowel Dis. 2015;21:2864-2872.

[8] Roggenbuck D, Vermeire S, Hoffman I, Reinhold D, Schierack P, Goihl A, et al. Evidence of Crohn's

For peer review only - http://bmjopen.bmj.com/site/about/guidelines.xhtml

For peer review only

disease-related anti-glycoprotein 2 antibodies in patients with celiac disease. Clin Chem Lab Med. 2015;53:1349-1357.

[9] Zhang S, Wu Z, Luo J, Ding X, Hu C, Li P, et al. Diagnostic Potential of Zymogen Granule Glycoprotein 2 Antibodies as Serologic Biomarkers in Chinese Patients With Crohn Disease. Medicine (Baltimore). 2015;94:e1654.

[10] Pavlidis P, Shums Z, Koutsoumpas AL, Milo J, Papp M, Umemura T, et al. Diagnostic and clinical significance of Crohn's disease-specific anti-MZGP2 pancreatic antibodies by a novel ELISA. Clin Chim Acta. 2015;441:176-181.

[11] Laass MW, Röber N, Range U, Noß L, Roggenbuck D, Conrad K. Loss and Gain of Tolerance to Pancreatic Glycoprotein 2 in Celiac Disease. PLoS One. 2015;10:e0128104.

[12] Papp M, Sipeki N, Tornai T, Altorjay I, Norman GL, Shums Z, et al. Rediscovery of the Anti-Pancreatic Antibodies and Evaluation of their Prognostic Value in a Prospective Clinical Cohort of Crohn's Patients: The Importance of Specific Target Antigens [GP2 and CUZD1]. J Crohns Colitis. 2015;9:659-668.

[13] Kohoutova D, Drahosova M, Moravkova P, Rejchrt S, Bures J. Anti-Outer membrane protein C and anti-glycoprotein 2 antibodies in inflammatory bowel disease and their association with complicated forms of Crohn's disease. BMC Gastroenterol. 2014;14:190.

[14] Gross S, Bakker SF, van Bodegraven AA, van Hoogstraten IM, Gelderman KA, Bouma G, et al. Increased IgA glycoprotein-2 specific antibody titres in refractory celiac disease. J Gastrointestin Liver Dis. 2014;23:127-133.

For peer review only - http://bmjopen.bmj.com/site/about/guidelines.xhtml

[15] Komorowski L, Teegen B, Probst C, Aulinger-Stöcker K, Sina C, Fellermann K, et al. Autoantibodies against exocrine pancreas in Crohn's disease are directed against two antigens: the glycoproteins CUZD1 and GP2. J Crohns Colitis. 2013;7:780-790.

[16] Bonaci-Nikolic B, Spuran M, Andrejevic S, Nikolic M. Autoantibodies to GP2, the major zymogen granule membrane glycoprotein, in patients with gluten-sensitive enteropathy: a possible serological trap. Clin Chim Acta. 2012;413:822-823.

[17] Pavlidis P, Romanidou O, Roggenbuck D, Mytilinaiou MG, Al-Sulttan F, Liaskos C, et al. Ileal inflammation may trigger the development of GP2-specific pancreatic autoantibodies in patients with Crohn's disease. Clin Dev Immunol. 2012;2012:640835.

[18] Bogdanos DP, Roggenbuck D, Reinhold D, Wex T, Pavlidis P, von Arnim U, et al. Pancreatic-specific autoantibodies to glycoprotein 2 mirror disease location and behaviour in younger patients with Crohn's disease. BMC Gastroenterol. 2012;12:102.

[19] Op De Beéck K, Vermeire S, Rutgeerts P, Bossuyt X. Antibodies to GP2, the major zymogen granule membrane glycoprotein, in inflammatory bowel diseases. Gut. 2012;61:162-4; author reply 164-165.

[20] Pavlidis P, Forbes A, Bogdanos DP. Antibodies to glycoprotein 2 (GP2) in patients with inflammatory bowel diseases from UK. Clin Chim Acta. 2011;412:1163-1164.

[21] Roggenbuck D, Hausdorf G, Martinez-Gamboa L, Reinhold D, Büttner T, Jungblut PR, et al. Identification of GP2, the major zymogen granule membrane glycoprotein, as the autoantigen of pancreatic antibodies in Crohn's disease. Gut. 2009;58:1620-1628.

[22] Satsangi J, Silverberg MS, Vermeire S, Colombel JF. The Montreal classification of inflammatory

For peer review only - http://bmjopen.bmj.com/site/about/guidelines.xhtml

For peer review only

bowel disease: controversies, consensus, and implications. Gut. 2006;55:749-753.

Figure legend

Fig 1. Flow chart of studies included in the meta-analysis.

Fig 2. The forest plots and the summary receiver operating characteristic curves of anti-glycoprotein 2 antibody (IgG) for CD. A: Sensitivity forest plot; B: Specificity forest plot; C: Summary Receiver Operating Characteristic.

Fig 3. The forest plots and the summary receiver operating characteristic curves of anti-glycoprotein 2 antibody (IgA) for CD. A: Sensitivity forest plot; B: Specificity forest plot; C: Summary Receiver Operating Characteristic.

Fig 4. The forest plots and the summary receiver operating characteristic curves of anti-glycoprotein 2 antibody (either IgG or IgA) for CD. A: Sensitivity forest plot; B: Specificity forest plot; C: Summary Receiver Operating Characteristic.

Fig 5. Funnel plot of the publication bias for the anti-glycoprotein 2 antibody (IgG and IgA either). ESS is the abbreviation of effective sample size.

For peer review only - http://bmjopen.bmj.com/site/about/guidelines.xhtml

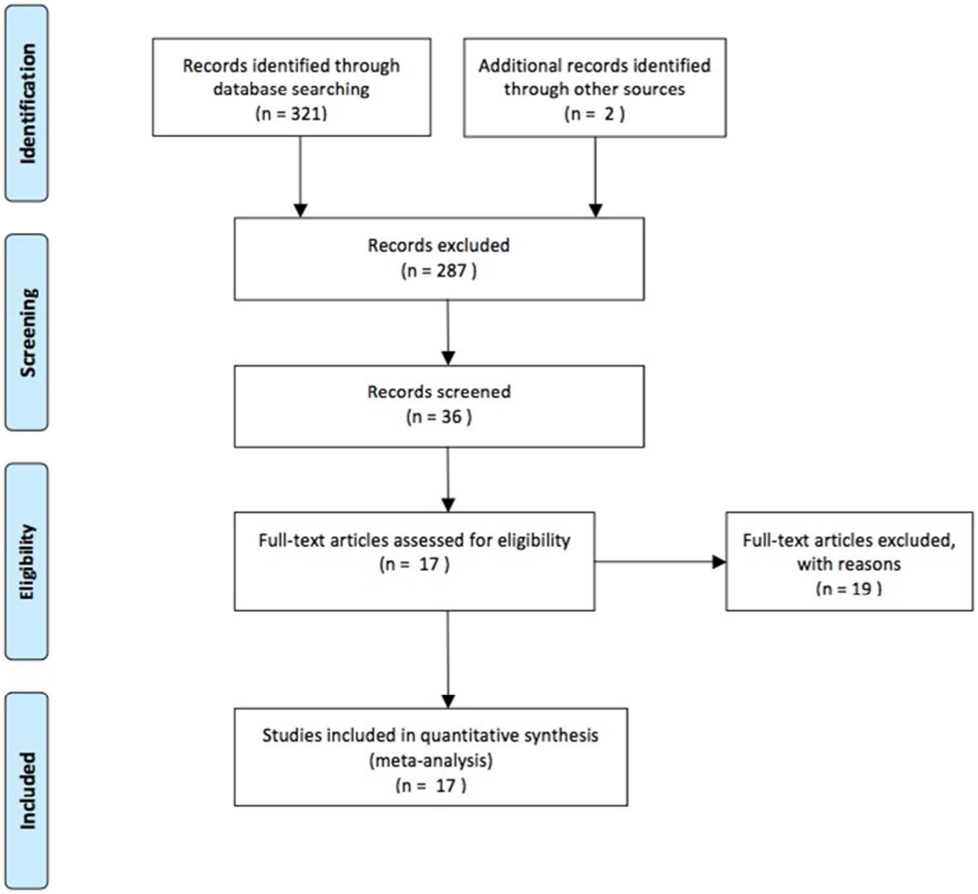

210x190mm (300 x 300 DPI)

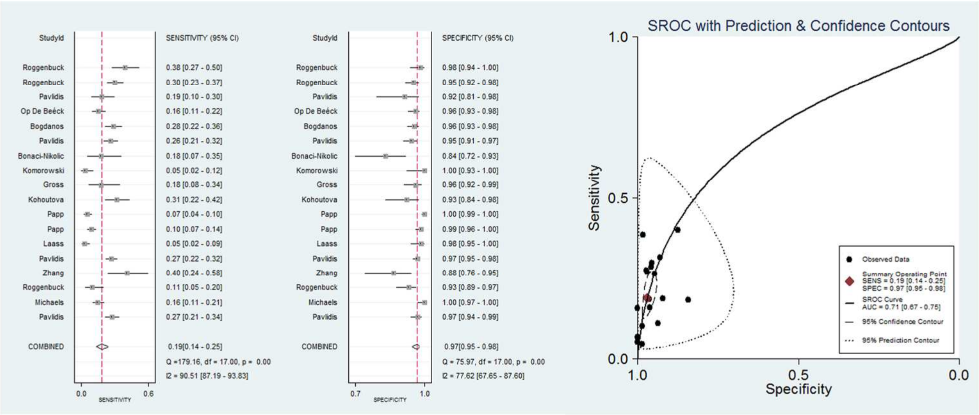

(A) (B) (C)

180x84mm (300 x 300 DPI)

For peer review only

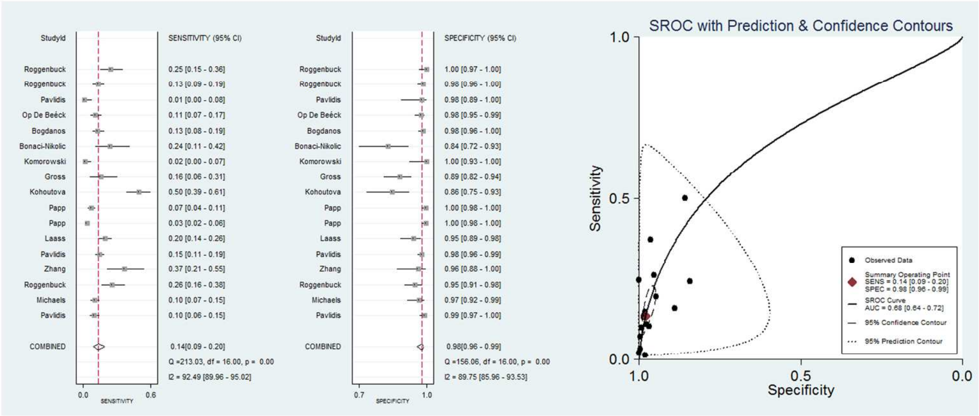

(A) (B) (C)

180x81mm (300 x 300 DPI)

For peer review only

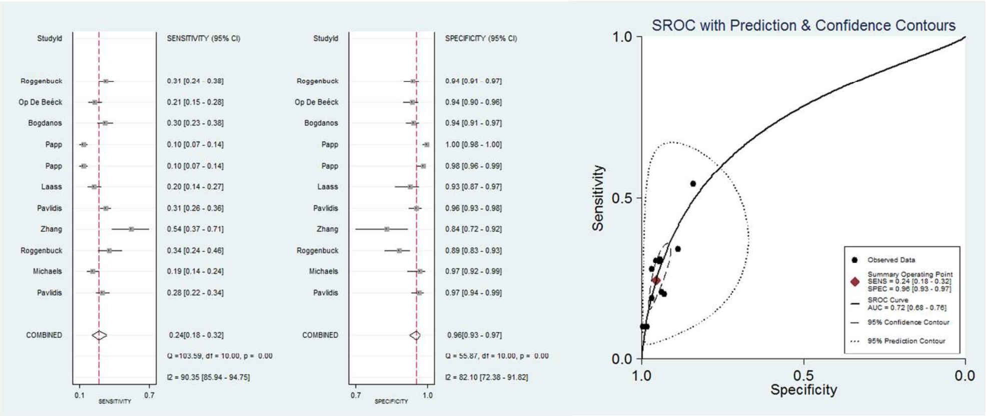

(A) (B) (C)

180x81mm (300 x 300 DPI)

er review only

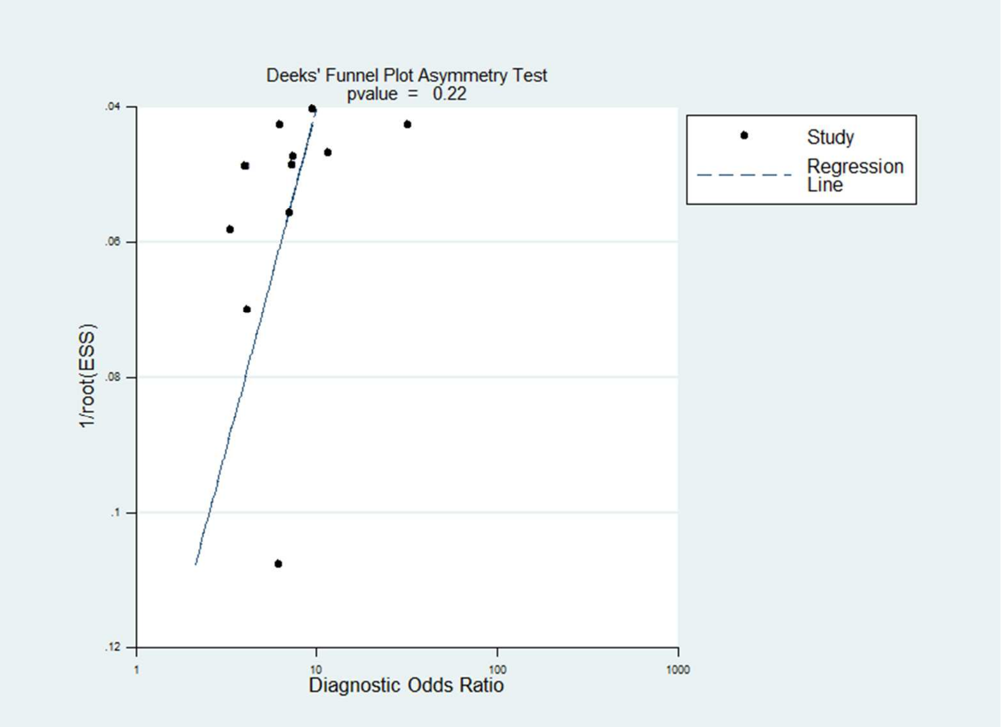

292x212mm (300 x 300 DPI)

For peer review only

The specific search algorithms of Pubmed

((anti-glycoprotein[All Fields] AND 2[All Fields] AND ("immunoglobulins"[MeSH Terms] OR "immunoglobulins"[All Fields] OR "antibody"[All Fields] OR "antibodies"[MeSH Terms] OR "antibodies"[All Fields])) OR (("glycoproteins"[MeSH Terms] OR "glycoproteins"[All Fields] OR "glycoprotein"[All Fields]) AND 2[All Fields] AND ("autoantibodies"[MeSH Terms] OR "autoantibodies"[All Fields])) OR (("autoantibodies"[MeSH Terms] OR "autoantibodies"[All Fields]) AND ("glycoproteins"[MeSH Terms] OR "glycoproteins"[All Fields] OR "glycoprotein"[All Fields]) AND 2[All Fields])) AND ("crohn disease"[MeSH Terms] OR ("crohn"[All Fields] AND "disease"[All Fields]) OR "crohn disease"[All Fields] OR ("crohn's"[All Fields] AND "disease"[All Fields]) OR "crohn's disease"[All Fields])

For peer review only

Supplementary Table 1 Characteristics of studies included in the meta-analysis of diagnostic performance of anti-glycoprotein 2 antibody in CD

Reference	Year	Region	Author	Manufacturer	Method	Ig subtype	Case	Control	TP	FP	FN	TN	QUADAS score
						IgG			58	7	154	242	
[6]	2016	United Kingdom	Pavlidis et al.	In-house kit	IIF	IgA	212	249	21	2	191	247	12
						either			59	8	153	241	
						IgG			35	0	189	136	
[7]	2015	Germany	Michaels et al.	Euroimmune	IIF	IgA	224	136	23	4	201	132	12
						either			42	4	182	132	
						IgG			8	11	65	158	
[8]	2015	Germany	Roggenbuck et al.	GA Generic Assays	ELISA	IgA	73	169	19	8	54	161	11
						either			25	19	48	150	
						IgG			14	7	21	49	
[9]	2015	China	Zhang et al.	GA Generic Assays	ELISA	IgA	35	56	13	2	22	54	11
						either			19	9	16	47	
						IgG	323	509	87	15	236	494	
[10]	2015	United Kingdom	Pavlidis et al.	Inova Diagnostics	ELISA	IgA			48	10	275	499	12
						either	323	294	99	13	224	281	
						IgG			8	2	166	124	
[11]	2015	Germany	Laass et al.	GA Generic Assays	ELISA	IgA	174	129	34	7	140	122	10
						either			35	9	139	117	

For peer review only

Supplementary Table 1 Characteristics of studies included in the meta-analysis of diagnostic performance of anti-glycoprotein 2 antibody in CD (continued)

Reference	Year	Region	Author	Manufacturer	Method	Ig subtype	Case	Control	TP	FP	FN	TN	QUADAS score
[12]	2015	Hungary	Papp et al.	GA Generic Assays	ELISA	IgG	266	283	27	4	239	279	12
						IgA			9	1	257	282	
						either			27	5	239	278	
				Euroimmune	IIF	IgG			18	0	248	283	
						IgA			19	1	247	282	
						either			27	1	239	282	
[13]	2014	Czech Republic	Kohoutova et al.	GA Generic Assays	ELISA	IgG	86	70	27	5	59	65	6
						IgA			43	10	43	60	
						either NA			/	/	/	/	
[14]	2014	Netherlands	Gross et al.	GA Generic Assays	ELISA	IgG	38	136	7	5	31	131	11
						IgA			6	15	32	121	
						either NA			/	/	/	/	
[15]	2013	Germany	Komorowski et al.	In-house kit	IIF	IgG	96	50	5	0	91	50	10
						IgA			2	0	94	50	
						either NA			/	/	/	/	
[16]	2012	Serbia	Bonaci-Nikolic et al.	GA Generic Assays	ELISA	IgG	33	57	6	9	27	48	11
						IgA			8	9	25	48	
						either NA			/	/	/	/	

For peer review only

Supplementary Table 1 Characteristics of studies included in the meta-analysis of diagnostic performance of anti-glycoprotein 2 antibody in CD (continued)

Reference	Year	Region	Author	Manufacturer	Method	Ig subtype	Case	Control	TP	FP	FN	TN	QUADAS score
						IgG			59	16	166	284	
[17]	2012	United Kingdom	Pavlidis et al.	GA Generic Assays	ELISA	IgA NA	225	300	/	/	/	/	11
						either NA			/	/	/	/	
						IgG			48	14	121	313	
[18]	2012	Germany	Bogdanos et al.	GA Generic Assays	ELISA	IgA	169	327	22	5	147	322	11
						either			51	18	118	309	
						IgG			26	11	138	282	
[19]	2012	Belgium	Op De Beéck et al.	GA Generic Assays	ELISA	IgA	164	293	18	7	146	286	12
						either			34	18	130	275	
						IgG			13	4	57	46	
[20]	2011	United Kingdom	Pavlidis et al.	GA Generic Assays	ELISA	IgA	70	50	1	1	69	49	6
						either NA			/	/	/	/	
						IgG			53	12	125	250	
[21]	2011	Germany	Roggenbuck et al.	In-house kit	ELISA	IgA	178	262	24	4	154	258	11
						either			55	15	123	247	
						IgG			28	2	45	116	
[22]	2009	Germany	Roggenbuck et al.	In-house kit	IIF	IgA	73	118	18	0	55	118	11
						either NA			/	/	/	/	

Note: IIF: indirect immunofluorescence; ELISA: enzyme-linked immuno sorbent assay; NA: not applicable; TP: true positive; FP: false positive; TN: true negative; FN: false negative.

For peer review only

PRISMA 2009 Checklist

Section/Topic	#	Checklist Item	Reported on Page #
**TITLE**			
Title	1	Identify the report as a systematic review, meta-analysis, or both.	1
**ABSTRACT**			
Structured summary	2	Provide a structured summary including, as applicable: background; objectives; data sources; study eligibility criteria, participants, and interventions; study appraisal and synthesis methods; results; limitations; conclusions and implications of key findings; systematic review registration number.	2
**INTRODUCTION**			
Rationale	3	Describe the rationale for the review in the context of what is already known.	4-5
Objectives	4	Provide an explicit statement of questions being addressed with reference to participants, interventions, comparisons, outcomes, and study design (PICOS).	5
**METHODS**			
Protocol and registration	5	Indicate if a review protocol exists, if and where it can be accessed (e.g., Web address), and, if available, provide registration information including registration number.	Not applicable
Eligibility criteria	6	Specify study characteristics (e.g., PICOS, length of follow-up) and report characteristics (e.g., years considered, language, publication status) used as criteria for eligibility, giving rationale.	5-6
Information sources	7	Describe all information sources (e.g., databases with dates of coverage, contact with study authors to identify additional studies) in the search and date last searched.	5
Search	8	Present full electronic search strategy for at least one database, including any limits used, such that it could be repeated.	5
Study selection	9	State the process for selecting studies (i.e., screening, eligibility, included in systematic review, and, if applicable, included in the meta-analysis).	5-6
Data collection process	10	Describe method of data extraction from reports (e.g., piloted forms, independently, in duplicate) and any processes for obtaining and confirming data from investigators.	6
Data items	11	List and define all variables for which data were sought (e.g., PICOS, funding sources) and any assumptions and simplifications made.	6
Risk of bias in individual studies	12	Describe methods used for assessing risk of bias of individual studies (including specification of whether this was done at the study or outcome level), and how this information is to be used in any data synthesis.	Not applicable
Summary measures	13	State the principal summary measures (e.g., risk ratio, difference in means).	6-7
Synthesis of results	14	Describe the methods of handling data and combining results of studies, if done, including measures of consistency (e.g., I^2) for each meta-analysis.	6-7

For peer review only

For peer review only - http://bmjopen.bmj.com/site/about/guidelines.xhtml

PRISMA 2009 Checklist

Section/Topic	#	Checklist Item	Reported on Page #
Risk of bias across studies	15	Specify any assessment of risk of bias that may affect the cumulative evidence (e.g., publication bias, selective reporting within studies).	7
Additional analyses	16	Describe methods of additional analyses (e.g., sensitivity or subgroup analyses, meta-regression), if done, indicating which were pre-specified.	7
**RESULTS**			
Study selection	17	Give numbers of studies screened, assessed for eligibility, and included in the review, with reasons for exclusions at each stage, ideally with a flow diagram.	8
Study characteristics	18	For each study, present characteristics for which data were extracted (e.g., study size, PICOS, follow-up period) and provide the citations.	8
Risk of bias within studies	19	Present data on risk of bias of each study and, if available, any outcome level assessment (see item 12).	Not applicable
Results of individual studies	20	For all outcomes considered (benefits or harms), present, for each study: (a) simple summary data for each intervention group (b) effect estimates and confidence intervals, ideally with a forest plot.	8-10
Synthesis of results	21	Present the main results of the review. If meta-analyses done, include for each, confidence intervals and measures of consistency.	8-10
Risk of bias across studies	22	Present results of any assessment of risk of bias across studies (see Item 15).	Not applicable
Additional analysis	23	Give results of additional analyses, if done (e.g., sensitivity or subgroup analyses, meta-regression [see Item 16]).	9-10
**DISCUSSION**			
Summary of evidence	24	Summarize the main findings including the strength of evidence for each main outcome; consider their relevance to key groups (e.g., healthcare providers, users, and policy makers).	11-13
Limitations	25	Discuss limitations at study and outcome level (e.g., risk of bias), and at review-level (e.g., incomplete retrieval of identified research, reporting bias).	13-14
Conclusions	26	Provide a general interpretation of the results in the context of other evidence, and implications for future research.	14
**FUNDING**			
Funding	27	Describe sources of funding for the systematic review and other support (e.g., supply of data); role of funders for the systematic review.	14

From: Moher D, Liberati A, Tetzlaff J, Altman DG, The PRISMA Group (2009). Preferred Reporting Items for Systematic Reviews and Meta-Analyses: The PRISMA Statement. PLoS Med 6(6): e1000097. doi:10.1371/journal.pmed1000097

For peer review only - http://bmjopen.bmj.com/site/about/guidelines.xhtml

PRISMA 2009 Checklist

For more information, visit: www.prisma-statement.org.

For peer review only

For peer review only - http://bmjopen.bmj.com/site/about/guidelines.xhtml
